# Dimensionless Groups by Entropic Similarity: I — Diffusion, Chemical Reaction and Dispersion Processes

**DOI:** 10.3390/e25040617

**Published:** 2023-04-05

**Authors:** Robert K. Niven

**Affiliations:** School of Engineering and Information Technology, The University of New South Wales, Canberra, ACT 2600, Australia; r.niven@adfa.edu.au

**Keywords:** dimensional analysis, entropic similarity, diffusion, chemical reaction, dispersion

## Abstract

Since the time of Buckingham in 1914, dimensional analysis and similarity arguments based on dimensionless groups have served as powerful tools for the analysis of systems in all branches of science and engineering. Dimensionless groups are generally classified into those arising from *geometric similarity*, based on ratios of length scales; *kinematic similarity*, based on ratios of velocities or accelerations; and *dynamic similarity*, based on ratios of forces. We propose an additional category of dimensionless groups based on *entropic similarity,* defined by ratios of (i) entropy production terms; (ii) entropy flow rates or fluxes; or (iii) information flow rates or fluxes. Since all processes involving work against friction, dissipation, diffusion, dispersion, mixing, separation, chemical reaction, gain of information or other irreversible changes are driven by (or must overcome) the second law of thermodynamics, it is appropriate to analyze them directly in terms of competing entropy-producing and transporting phenomena and the dominant entropic regime, rather than indirectly in terms of forces. In this study, entropic groups are derived for a wide variety of diffusion, chemical reaction and dispersion processes relevant to fluid mechanics, chemical engineering and environmental engineering. It is shown that many dimensionless groups traditionally derived by kinematic or dynamic similarity (including the Reynolds number) can also be recovered by entropic similarity—with a different entropic interpretation—while many new dimensionless groups can also be identified. The analyses significantly expand the scope of dimensional analysis and similarity arguments for the resolution of new and existing problems in science and engineering.

## 1. Introduction

Since the seminal work of Buckingham [1], built on the insights of many predecessors [2,3,4,5,6,7,8,9,10], dimensional analysis and similarity arguments based on dimensionless groups have provided a powerful tool—and in many cases, the most important tool—for the analysis of physical, chemical, biological, geological, environmental, astronomical, mechanical and thermodynamic systems, especially those involving fluid mechanics. The dimensionless groups obtained are usually classified into those arising from *geometric similarity*, based on ratios of length scales (or areas or volumes); *kinematic similarity*, based on ratios of velocities or accelerations; and *dynamic similarity*, based on ratios of forces [11,12,13,14,15,16,17,18]. Thus, for example, the Reynolds number [19] is generally interpreted by dynamic similarity as:

(1) $$\begin{array}{c} Re=\frac{\mathrm{inertial}\;\mathrm{force}}{\mathrm{viscous}\;\mathrm{force}}=\frac{F_{I}}{F_{\nu }}∼\frac{\rho d^{3}\left(U^{2}/d\right)}{\mu \left(U/d\right)d^{2}}=\frac{\rho Ud}{\mu }=\frac{Ud}{\nu } \end{array}$$

where ∼ indicates “of the order of” (discarding numerical constants), ρ is the fluid density [SI units: kg m${}^{-3}$], μ is the dynamic viscosity [Pa s], ν is the kinematic viscosity [m${}^{2}$ s${}^{-1}$], *U* is a velocity scale [m s${}^{-1}$] and *d* is an applicable length scale [m]. In common with many dimensionless groups, Equation (1) provides an identifier of the flow regime, reflecting the fact that viscous forces—causing laminar flow—are dominant at Reynolds numbers below some critical value $Re_{c}$, while inertial forces—leading to flows of increasingly turbulent character—will be dominant above this critical value.

The aim of this work is to provide a new interpretation for a large class of dimensionless groups based on the principle of *entropic similarity*, involving ratios of entropic terms. Since all processes involving work against friction, dissipation, diffusion, dispersion, mixing, separation, chemical reaction, gain of information or other irreversible changes are driven by (or must overcome) the second law of thermodynamics, it is appropriate to analyze these processes directly in terms of competing entropy-producing and transporting phenomena and the dominant entropic regime, rather than indirectly in terms of forces. As will be shown, an entropic perspective enables the reinterpretation of many known dimensionless groups—including the Reynolds number (1)—as well as the formulation of many new groups. These significantly expand the scope of dimensional analysis and similarity arguments for the resolution of new and existing problems.

We further note that while the energetic formulation of thermodynamics developed over the last 150 years—especially by Gibbs [20]—has been of tremendous utility, its underlying basis is entropic, as was recognized by Gibbs and other prominent researchers [21,22,23,24,25,26,27,28,29,30,31]. Such researchers understood that “dissipation”, the irreversible loss of organized energy or information, is not a driving force in its own right but a consequence of the second law of thermodynamics. It is therefore appropriate that dissipative phenomena be expressed in terms of entropic rather than energetic quantities. In this manner, the *quality* or *temper* of the degradation—expressed by a thermodynamic integration factor such as 1/T, where *T* is the absolute temperature [K]—is explicitly recognized [27].

This work is set out as follows. Firstly, in Section 2, the theoretical foundations of the entropic perspective are examined in detail. This includes the combinatorial and information-theoretic definitions of entropy, the maximum entropy method, the thermodynamic entropy balance equation and the entropy production in global and local forms. The importance of dimensional arguments and current definitions of similarity are examined in Section 3, following which the principle of entropic similarity is established, with three interpretations. A number of entropy-producing and transporting phenomena relevant to fluid mechanics, chemical and process engineering, environmental engineering and cosmology are then examined in detail, divided into diffusion and chemical reaction processes (Section 4), a variety of dispersion mechanisms (Section 5) and diffusion in the universe (Section 6). Dimensionless groups are derived for these phenomena by entropic similarity and compared to the traditional groups obtained by other methods, to examine their similarities and differences and reveal some important new insights. The conclusions are given in Section 7.

We note that this study spans several branches of science and engineering, and some clashes of standard symbols cannot be avoided; where present, these are explicitly mentioned. The SI units of each quantity are also included—in the author’s experience, these are far more informative for both teaching purposes and dimensional analysis than the widely used “dimensions” (M,L,t,T) notation.

## 2. Theoretical Foundations

### 2.1. Dimensionless Entropy and Information

We first review the entropy concept and its connections to combinatorics and information theory. The (dimensionless) discrete entropy function was given by Shannon [32]:

(2) $$\begin{array}{c} H_{\mathrm{Sh}}=-\sum_{i=1}^{n}p_{i}\ln p_{i} \end{array}$$

where $p_{i}$ is the probability of the *i*th outcome or category, from *n* such categories. For a system with unequal prior probabilities $q_{i}$ for each category, it is necessary to adopt the relative entropy function or negative Kullback–Leibler divergence [25,33,34]:

(3) $$H=-\sum_{i=1}^{n}p_{i}\ln\frac{p_{i}}{q_{i}}$$

For a continuous variable $x\in R^{m}$ with m∈N, both (2) and (3) converge to the continuous relative entropy [25,35]:

(4) $$H=-\int_{\Omega_{x}}dx\;p\left(x\right)\ln\frac{p(x)}{q(x)}$$

where $\Omega_{x}$ is the domain of x, $dx=dx_{1}\cdots dx_{m}$, and p(x) and q(x) are respectively the posterior and prior probability density functions (pdfs).

The discrete entropy functions (2) and (3) can be derived from the axiomatic properties of a measure of uncertainty [32,34,36]. In information theory, both are commonly rewritten using base 2 logarithms to give measures of information, expressed in binary digits or “bits” [37]. Equations (2) and (3) can also be obtained from the combinatorial definition of entropy [38]:

(5) $$H=\frac{1}{N}\ln P$$

where P is the governing probability distribution of the system, representing an allocation scheme for *N* entities. For a system composed of distinguishable entities allocated to distinguishable states, P is given by the multinomial distribution $P=N!\prod_{i=1}^{s}q_{i}^{n_{i}}/n_{i}!$, where $n_{i}$ entities are allocated to the *i*th state and $\sum_{i=1}^{s}n_{i}=N$. In the asymptotic limits N→∞ and $n_{i}/N\to p_{i}$, Equation (5) converges to the discrete relative entropy (3) [38,39,40]. Alternative entropy functions for different governing distributions [41,42,43,44] can be derived from (5).

In the maximum entropy (MaxEnt) method, the user maximizes the appropriate entropy function (2)–(4), subject to its (R+1) moment constraints, to give the inferred distribution of the system and its maximum entropy, respectively [23,24,25]:

(6) $$\begin{array}{c} p_{i}^{*}=\frac{q_{i}}{Z}\exp\left(-\sum_{r=0}^{R}\lambda_{r}f_{ri}\right)\;\mathrm{or}\;p^{*}\left(x\right)=\frac{q(x)}{Z}\exp\left(-\sum_{r=0}^{R}\lambda_{r}f_{r}\left(x\right)\right) \end{array}$$

(7) $$\begin{array}{c} H^{*}=\ln Z+\sum_{r=1}^{R}\lambda_{r}\langle f_{r}\rangle \end{array}$$

where ${}^{*}$ denotes the inferred state, *Z* is the partition function and, for the *r*th category, $f_{ri}$ or $f_{r}\left(x\right)$ is the local category value, $\langle f_{r}\rangle$ is the expected category value and $\lambda_{r}$ is the Lagrangian multiplier. In the Boltzmann interpretation, Equation (6) gives the most probable distribution of the system. The entire body of thermodynamics can readily be derived by this method based on the thermodynamic entropy $S=k_{B}H^{*}$, where $k_{B}$ is the Boltzmann constant [J K${}^{-1}$] [23,24,25,26,27]. The MaxEnt method has also been used to determine the stationary state of many systems beyond thermodynamics, including hydraulic, hydrological, geological, biological, ecological and financial systems as well as water distribution, electrical and transport networks [25,34,45,46,47,48,49,50,51].

### 2.2. Thermodynamic Entropy Balance and Entropy Production

We now consider flows of the thermodynamic entropy *S* [J K${}^{-1}$]. For an open system, the balance equation for thermodynamic entropy in a macroscopic control volume is:

(8) $$\begin{array}{c} \frac{DS_{FV(t)}}{Dt}=\frac{\partial S_{CV}}{\partial t}+F_{S,f}^{out}-F_{S,f}^{in} \end{array}$$

where *t* is time, FV is the fluid volume, CV is the control volume, D/Dt is the substantial derivative and $F_{S,f}^{out}$ and $F_{S,f}^{in}$ are respectively the flow rates of *S* out of and into the control volume by fluid flow [J K${}^{-1}$ s${}^{-1}$]. By the de Donder method, the substantial derivative in (8) can be separated into internally and externally driven rates of change, respectively:

(9) $$\begin{array}{c} \frac{DS_{FV(t)}}{Dt}=\frac{D_{i}S_{FV(t)}}{Dt}+\frac{D_{e}S_{FV(t)}}{Dt} \end{array}$$

The first term on the right of Equation (9) is the rate of entropy production, commonly denoted $\dot{\sigma }$ [J K${}^{-1}$ s${}^{-1}$]. The second term on the right of Equation (9) is the total rate of change of entropy due to non-fluid flows, such as heat or chemical species:

(10) $$\begin{array}{c} \frac{D_{e}S_{FV(t)}}{Dt}=F_{S,nf}^{in}-F_{S,nf}^{out} \end{array}$$

where $F_{S,nf}^{out}$ and $F_{S,nf}^{in}$ are respectively the outward and inward non-fluid flow rates of *S* [J K${}^{-1}$ s${}^{-1}$]. Combining Equations (8)–(10) and rearrangement gives:

(11) $$\begin{array}{c} \dot{\sigma }=\frac{\partial S_{CV}}{\partial t}+F_{S,f}^{out}-F_{S,f}^{in}+F_{S,nf}^{out}-F_{S,nf}^{in}=\frac{\partial S_{CV}}{\partial t}+F_{S,tot}^{net}\geq 0 \end{array}$$

where $F_{S,tot}^{net}$ is the net total outwards entropy flow rate [J K${}^{-1}$ s${}^{-1}$].

From the second law of thermodynamics, the entropy production (11) must be non-negative. In contrast, the rate of change of entropy within the system $\partial S_{CV}/\partial t$ can take any sign but will vanish at the steady state. The entropy production therefore represents the irreversible rate of increase in entropy of the universe due to the system; at steady state, this is equal to the rate at which the system exports entropy to the rest of the universe. Unfortunately, there is still considerable confusion in the literature between the rate of entropy production of a system $\dot{\sigma }$ and its rate of change of entropy $\partial S_{CV}/\partial t$.

For an integral control volume commonly examined in fluid mechanics, the entropy balance equation is given by Reynolds’ transport theorem [52,53,54]:

(12) $$\begin{array}{c} \frac{DS_{FV(t)}}{Dt}=\frac{\partial }{\partial t}\underset{CV}{\;\int \int \int \;}\rho s\;dV+{∯}_{CS}\rho su\cdot ndA=\underset{CV}{\;\int \int \int \;} [\frac{\partial }{\partial t}\rho s+\nabla \cdot \rho su]dV \end{array}$$

where CS is the control surface, *s* is the specific entropy (per unit mass of fluid) [J K${}^{-1}$ kg${}^{-1}$], u is the fluid velocity [m s${}^{-1}$], dV is a volume element [m${}^{3}$], dA is an area element [m${}^{2}$], n is an outwardly directed unit normal [-] and ∇ is the Cartesian nabla operator [m${}^{-1}$]. From (9), we can write:

(13) $$\begin{array}{c} \frac{DS_{FV(t)}}{Dt}=\dot{\sigma }-\underset{FS(t)}{∯}j_{S}\cdot ndA \end{array}$$

where $j_{S}$ is the outward non-fluid entropy flux [J K${}^{-1}$ m${}^{-2}$ s${}^{-1}$] and FS(t) is the fluid surface coincident with the control surface at time *t*. Combining Equations (12) and (13) and rearrangement gives [29,31,55,56,57,58,59]:

(14) $$\begin{array}{c} \dot{\sigma }=\underset{CV}{\;\int \int \int \;}\frac{\partial \rho s}{\partial t}dV+\underset{CS}{∯}J_{S}\cdot ndA=\underset{CV}{\;\int \int \int \;} [\frac{\partial }{\partial t}\rho s+\nabla \cdot J^{S}]dV\geq 0 \end{array}$$

where $J^{S}=j_{S}+\rho su$ is the total outward entropy flux [J K${}^{-1}$ m${}^{-2}$ s${}^{-1}$].

To establish a local entropy balance equation, we first define the rate of entropy production per unit volume $\hat{\dot{\sigma }}$ [J K${}^{-1}$ m${}^{-3}$ s${}^{-1}$] by the integral:

(15) $$\begin{array}{c} \dot{\sigma }=\underset{CV}{\;\int \int \int \;}\hat{\dot{\sigma }}dV \end{array}$$

Applying the fundamental lemma of the calculus of variations to Equation (14), we can then extract the differential equation for the local entropy production [31,55,56,57,58]:

(16) $$\begin{array}{c} \hat{\dot{\sigma }}=\frac{\partial }{\partial t}\rho s+\nabla \cdot J_{S}\geq 0 \end{array}$$

From the second law, $\hat{\dot{\sigma }}$ is non-negative, whereas the local rate ∂s/∂t can take any sign. At local steady state ∂(ρs)/∂t=0 and $\hat{\dot{\sigma }}=\nabla \cdot J_{S}\geq 0$.

## 3. Dimensionless Groups and the Principle of Entropic Similarity

A *dimensionless group* is a unitless (dimensionless) parameter used to represent an attribute of a physical system, independent of the system of units used. By the late 19th century, researchers had established the concept of *similarity* or *similitude* between a system (prototype) and its model based on matching dimensionless groups, so that their mechanical or physical properties would be equivalent [6,8]. Such dimensional scaling offers the advantage of smaller-scale models, greatly simplifying the experimental requirements. The formal method of *dimensional analysis* was then developed to extract the functional dependencies of a physical system from its list of parameters [1]. This also enables order reduction, reducing the number of parameters by the number of dimensions. For over a century, these dimensional methods have been recognized as powerful tools—and in many cases, the primary tools—for the analysis of a wide range of systems across all branches of science and engineering [60,61,62,63,64,65,66].

More recently, dimensional analysis has been found to have strong connections to group theory, in particular to continuous (Lie) groups arising from symmetries in the governing equations of a system [63,67,68,69,70,71,72]. This includes a deep connection to the one-parameter Lie group of point scaling transformations [71,73,74,75]. Recently, this was shown to enable the non-dimensionalization of a differential equation based on its intrinsic dimensions [76]. Lie symmetry is therefore an important invariance property of a differential equation, subsuming other invariances such as dimensional scaling, invariance to fixed coordinate displacements, rotations or reflections, and Galilean invariance [76].

Dimensionless groups—commonly labeled Π—are usually classified as follows [11,12,13,14,15,16,18]:

(i) Those arising from *geometric similarity*, based on ratios of length scales $\ell_{i}$ [m] or associated areas or volumes:
  (17) $$\begin{array}{c} \Pi_{\mathrm{geom}}=\frac{\ell_{1}}{\ell_{2}}\;\;\mathrm{or}\;\;\Pi_{\mathrm{geom}}=\frac{\ell_{1}^{2}}{\ell_{2}^{2}}\;\;\mathrm{or}\;\;\Pi_{\mathrm{geom}}=\frac{\ell_{1}^{3}}{\ell_{2}^{3}} \end{array}$$
(ii) Those arising from *kinematic similarity*, based on ratios of magnitudes of velocities $U_{i}$ [m s${}^{-1}$] or accelerations $a_{i}$ [m s${}^{-2}$]:
  (18) $$\begin{array}{c} \Pi_{\mathrm{kinem}}=\frac{U_{1}}{U_{2}}\;\;\mathrm{or}\;\;\Pi_{\mathrm{kinem}}=\frac{a_{1}}{a_{2}} \end{array}$$
(iii) Those arising from *dynamic similarity*, based on ratios of magnitudes of forces $F_{i}$ [N]:
  (19) $$\begin{array}{c} \Pi_{\mathrm{dynam}}=\frac{F_{1}}{F_{2}} \end{array}$$

Such dimensionless groups are generally obtained by three methods: (i) assembled *a priori* by an assessment of the dominant phenomena of a system; (ii) extracted from a list of parameters by dimensional analysis; or (iii) obtained by non-dimensionalization of the governing differential equation(s) for the system. It is generally accepted that for a model and a system (prototype) to exhibit the same physics—expressed in terms of identical values of dimensionless groups—they must satisfy the conditions of geometric, kinematic and dynamic similarity.

Here, we propose an additional category of dimensionless groups based on *entropic similarity*, involving ratios of entropic terms. This enables the direct analysis of phenomena involving friction, dissipation, diffusion, dispersion, mixing, separation, chemical reaction, gain of information or other irreversible changes governed by the second law of thermodynamics, as well as entropy transport processes. At present, these processes are usually indirectly examined based on dynamic similarity (19), requiring their conversion into forces, which for many phenomena can be rather contrived. We distinguish three variants of entropic groups:

(i) Those defined by ratios of global (14) or local (16) entropy production terms:
  (20) $$\begin{array}{c} \Pi_{\mathrm{entrop}}=\frac{{\dot{\sigma }}_{1}}{{\dot{\sigma }}_{2}}\;\;\mathrm{or}\;\;{\hat{\Pi }}_{\mathrm{entrop}}=\frac{{\hat{\dot{\sigma }}}_{1}}{{\hat{\dot{\sigma }}}_{2}} \end{array}$$
  where Π represents a global or summary dimensionless group and $\hat{\Pi }$ a local group.
(ii) Those defined by ratios of global flow rates of thermodynamic entropy, such as in (11), or by components or magnitudes of their local fluxes, such as in (16):
  (21) $$\begin{array}{c} \Pi_{\mathrm{entrop}}=\frac{F_{S,1}}{F_{S,2}}\;\;\mathrm{or}\;\;{\hat{\Pi }}_{\mathrm{entrop}}\left(n\right)=\frac{j_{S_{1}}\cdot n}{j_{S_{2}}\cdot n}\;\;\mathrm{or}\;\;{\hat{\Pi }}_{\mathrm{entrop}}=\frac{\left|\right|j_{S_{1}}\left|\right|}{\left|\right|j_{S_{2}}\left|\right|} \end{array}$$
  where n is a unit normal and $\left||a|\right|=\sqrt{a^{⊤}a}$ is the Euclidean norm for vector a.
(iii) Those defined by an information-theoretic threshold, given locally by the ratio of the information flux carried by the flow $j_{I,\mathrm{flow}}$ [bits m${}^{-2}$ s${}^{-1}$] to that transmitted by a carrier of information $j_{I,\mathrm{signal}}$ [bits m${}^{-2}$ s${}^{-1}$] in the direction of a given unit normal n:
  (22) $$\begin{array}{c} {\hat{\Pi }}_{\mathrm{info}}\left(n\right)=\frac{j_{I,\mathrm{flow}}\cdot n}{j_{I,\mathrm{signal}}\cdot n}=\frac{\rho_{I,\mathrm{flow}}\;u_{\mathrm{flow}}\cdot n}{\rho_{I,\mathrm{signal}}\;u_{\mathrm{signal}}\cdot n} \end{array}$$
  In this perspective, flows in which the information flux of the fluid exceeds that of a signal (${\hat{\Pi }}_{\mathrm{info}}>1$) will experience a different information-theoretic flow regime to those in which the signal flux dominates (${\hat{\Pi }}_{\mathrm{info}}<1$). In Equation (22), each information flux is further reduced to the product of an information density $\rho_{I}$ [bits m${}^{-3}$] and the corresponding fluid velocity $u_{\mathrm{flow}}$ or signal velocity $u_{\mathrm{signal}}$ [m s${}^{-1}$]. Making the strong assumption that the two information densities are comparable, Equation (22) reduces to the local or summary kinematic definitions:
  (23) $$\begin{array}{c} {\hat{\Pi }}_{\mathrm{info}}\left(n\right)=\frac{u_{\mathrm{flow}}\cdot n}{u_{\mathrm{signal}}\cdot n},\;\Pi_{\mathrm{info}}=\frac{U_{\mathrm{flow}}}{U_{\mathrm{signal}}} \end{array}$$
  where $U_{\mathrm{flow}}$ and $U_{\mathrm{signal}}$ are representative flow and signal velocities [m s${}^{-1}$].

In the following sections, we examine a succession of natural processes relating to diffusion, chemical reaction and dispersion, involving competition between various entropy-producing and/or entropy-transporting phenomena. Within each class of processes, the principle of entropic similarity is invoked to construct families of dimensionless groups. These are compared to the well-known groups formed by dynamic similarity, to examine the similarities and contrasts between the two approaches and reveal several important new insights.

## 4. Diffusion and Chemical Reaction Processes

### 4.1. Independent Diffusion Processes

#### 4.1.1. Practical Diffusion Relations

We first consider diffusion processes (often termed *transport phenomena*) acting independently, in which a gradient in a physical field induces a flux of the corresponding (conjugate) physical quantity. Usually, the term *diffusion* is applied to processes acting at molecular scales, while *dispersion* is applied to analogous mixing processes acting at microscopic to macroscopic scales. All diffusion and dispersion processes are irreversible, driven by or causing an increase in thermodynamic entropy due to mixing.

The diffusion of heat, momentum, chemical species or charge due to the random motions of molecules, for binary species and in the absence of electromagnetic effects (thus for conservative fields), anisotropy or cross-phenomenological processes, are commonly analyzed by the following practical or empirical relations [16,28,31,58,77,78,79,80,81,82]:

(24) $$\begin{array}{ccc} j_{Q} & =-k\nabla T & \;\;\;\;\;\;\;\;\;\;\;\;\;\;\;\;\;\;\mathrm{Fourier}’s\;\mathrm{law} \end{array}$$

(25) $$\begin{array}{ccc} \tau & =-\mu \left(\nabla u+{\left(\nabla u\right)}^{⊤}\right)=-\rho \nu \left(\nabla u+{\left(\nabla u\right)}^{⊤}\right)\; & \;\;\;\;\mathrm{Newton}’s\;\mathrm{law} \end{array}$$

(26) $$\begin{array}{ccc} j_{c} & =-D_{c}\nabla C_{c}=-D_{c}\nabla \left(\rho m_{c}\right) & \;\;\;\;\;\;\;\;\;\mathrm{Fick}’s\;\mathrm{first}\;\mathrm{law}\;\left(\mathrm{binary}\right) \end{array}$$

(27) $$\begin{array}{ccc} i_{k} & =-\kappa_{k}\nabla \Phi =\kappa_{k}\;E & \;\;\;\;\;\;\;\;\;\;\;\;\;\;\;\;\;\mathrm{Ohm}’s\;\mathrm{law} \end{array}$$

These are based on the following quantities. *Fluxes*: $j_{Q}$ is the heat flux [J m${}^{-2}$ s${}^{-1}$], τ is the momentum flux or viscous stress tensor [Pa] (defined positive in compression; [58]), $j_{c}$ is the molar flux of the *c*th chemical species [(mol species) m${}^{-2}$ s${}^{-1}$] (relative to the molar average velocity $\langle u\rangle =\sum_{c}C_{c}u_{c}/\sum_{c}C_{c}$) and $i_{k}$ is the charge flux or current density of the *k*th charged species (defined in the direction of positive ion flow) [C m${}^{-2}$ s${}^{-1}$ = A m${}^{-2}$], for which $i=\sum_{k}i_{k}$ is the total charge flux [A m${}^{-2}$]. *Fields*: *T* is the absolute temperature [K], u is the (mass-average) fluid velocity [m s${}^{-1}$], $u_{c}$ is the (mass-average) velocity of species *c* [m s${}^{-1}$], $C_{c}$ is the molar concentration of chemical species *c* per unit volume [(mol species) m${}^{-3}$], $m_{c}=C_{c}/\rho$ is the molality (specific molar concentration) of chemical species *c* [(mol species) kg${}^{-1}$], ρ is the fluid density [kg m${}^{-3}$], Φ is the electrical (electrostatic) potential [V = J C${}^{-1}$] and E=−∇Φ is the electric field vector [V m${}^{-1}$]. *Diffusion parameters*: *k* is the thermal conductivity [J K${}^{-1}$ m${}^{-1}$ s${}^{-1}$], μ is the dynamic viscosity [Pa s], ν is the kinematic viscosity or momentum diffusion coefficient [m${}^{2}$ s${}^{-1}$], $D_{c}$ is the diffusion coefficient for the *c*th chemical species [m${}^{2}$ s${}^{-1}$] and $\kappa_{k}$ is the electrical conductivity or specific conductance for the *k*th charged species [A V${}^{-1}$ m${}^{-1}$]. *Vector quantities*: $x={\left[x,y,z\right]}^{⊤}$ is the Cartesian position vector [m], $\nabla ={\left[\partial /\partial x,\partial /\partial y,\partial /\partial z\right]}^{⊤}$ is the Cartesian gradient operator [m${}^{-1}$] (using the ∂(↓)/∂(→) convention for vector gradients such as ∇u), and ${}^{⊤}$ is the vector or matrix transpose. In engineering mechanics, heat diffusion is commonly termed *conduction*, while in electrical engineering, the diffusion of charge by Ohm’s law is termed *drift* to distinguish it from the diffusion of charge carriers under Fick’s law (26). Many authors refer to fluxes as *flux densities* and to diffusion coefficients as *diffusivities*.

We emphasize that (24)–(27) are first-order correlations, and more complicated forms are required in many circumstances, e.g., for non-Newtonian fluids, inhomogeneous materials or multicomponent or unsteady processes [81,83]. In anisotropic media, it may also be necessary to redefine $k,\nu ,D_{c}$ or $\kappa_{k}$ as a second-order tensor [28,56,58].

Fick’s first law (26) is expressed in terms of the density of the *c*th species. It is defined here using molar fluxes and concentrations but can be written in terms of mass quantities [58]. Fourier’s and Newton’s laws can be rewritten respectively in terms of the energy or momentum density [58,82]:

(28) $$\begin{array}{ccc} j_{Q} & =-\alpha \nabla (\rho c_{p}T) & \;\;\;\;\;\;\;\;\;\;\;\;\;\;\;\;\;\;\;\;\;\;\;\;\;\;\;\;\;\;\mathrm{Fourier}’s\;\mathrm{law}\;\left(\mathrm{energy}\right) \end{array}$$

(29) $$\begin{array}{ccc} \tau & =-\nu (\nabla \left(\rho u\right)+{\left[\nabla \left(\rho u\right)\right]}^{⊤}) & \mathrm{Newton}’s\;\mathrm{law}\;\left(\mathrm{momentum}\right) \end{array}$$

where α is the thermal diffusion coefficient [m${}^{2}$ s${}^{-1}$] and $c_{p}$ is the specific heat capacity of the fluid at constant pressure [J K${}^{-1}$ kg${}^{-1}$]. Ohm’s law can also be written in terms of the charge density of the *k*th charged species [84,85]:

(30) $$\begin{array}{ccc} i_{k} & =-D_{k}\nabla \left(\frac{z_{k}^{2}F^{2}C_{k}\Phi }{RT}\right)=-D_{k}\nabla \left(\frac{q_{k}^{2}n_{k}\Phi }{k_{B}T}\right)\; & \mathrm{Ohm}’s\;\mathrm{law}\;\left(\mathrm{charge}\right) \end{array}$$

where *F* is the Faraday constant [C (mol charge)${}^{-1}$], *R* is the ideal gas constant [J K${}^{-1}$ (mol species)${}^{-1}$] and, for the *k*th charged species, $C_{k}$ is the molar concentration [(mol species) m${}^{-3}$], $D_{k}$ is the diffusion coefficient [m${}^{2}$ s${}^{-1}$], $n_{k}$ is the number density [m${}^{-3}$], $q_{k}$ is the charge [C], and $z_{k}$ is the charge number (valency) [(mol charge) (mol species)${}^{-1}$]. Equation (30) builds upon a number of electrochemical relations (see Appendix A). The first form of Ohm’s law in (30) is written on a molar basis, appropriate (with $C_{k}=\rho m_{k}$) for charged species in aqueous solution, while the second form accords with the individual carrier formulation used in electrical engineering to analyze the drift of electrons or holes in a conductor.

As evident, the density formulations of the diffusion Equations (26) and (28)–(30) contain diffusion coefficients ($\alpha ,\nu ,D_{c}$ and $D_{k}$) with SI units of m${}^{2}$ s${}^{-1}$. Comparing (24) to (28) and (27) to (30), we obtain $\alpha ≃k/\rho c_{p}$ and $D_{k}≃RT\kappa_{k}/z_{k}^{2}F^{2}C_{k}=k_{B}T\kappa_{k}/q_{k}^{2}n_{k}$, with strict equality for spatially homogeneous properties. Each diffusion coefficient can be further expressed as a function of molecular properties for different states of matter [58,83,86].

#### 4.1.2. Thermodynamic Diffusion Relations

Although of tremendous utility in practical applications, the above diffusion laws can be reformulated in terms of thermodynamic gradients. A thermodynamic framework has the advantage of providing a sound theoretical foundation and can incorporate more phenomena (including cross-phenomenological effects), but it does come at the expense of dimensionally more complicated parameters. As will be shown, it can also be connected directly to the entropy production. The four laws become [16,28,31,58,77,87]:

(31) $$\begin{array}{ccc} j_{Q} & =\alpha^{\prime }\nabla \frac{1}{T} & \;\;\;\;\;\;\;\;\;\;\;\;\;\;\;\;\;\;\;\;\;\;\;\;\;\;\;\;\;\;\;\;\;\;\;\;\;\;\;\;\;\;\;\;\;\;\;\;\;\;\;\;\;\;\;\;\;\;\;\;\mathrm{Fourier}’s\;\mathrm{law}\;\left(\mathrm{thermodynamic}\right) \end{array}$$

(32) $$\begin{array}{cc} \begin{array}{cc} \tau & =-\mu (\nabla u+{\left(\nabla u\right)}^{⊤})-\lambda \;\delta \;\nabla \cdot u\\ & =-2\mu e-\lambda \;\delta \;\mathrm{tr}(e) \end{array} & \;\;\;\;\;\;\;\;\;\;\;\;\;\;\;\;\;\;\;\;\;\;\;\;\;\;\;\;\;\;\;\;\;\;\;\;\;\;\;\;\mathrm{Newton}’s\;\mathrm{law}\;\left(\mathrm{thermodynamic}\right) \end{array}$$

(33) $$\begin{array}{ccc} j_{c} & =-D_{c}^{\prime }\nabla \frac{\mu_{c}}{T} & \;\;\;\;\;\;\;\;\;\;\;\;\;\;\;\;\;\;\;\;\;\;\;\;\;\;\;\;\;\;\;\;\;\;\;\;\;\;\;\;\;\;\;\;\;\;\;\;\;\;\;\;\;\;\;\;\;\;\;\;\mathrm{Fick}’s\;\mathrm{first}\;\mathrm{law}\;(\mathrm{binary},\;\mathrm{thermodynamic}) \end{array}$$

(34) $$\begin{array}{ccc} i_{k} & =-{D_{k}}^{\prime }\frac{\nabla \Phi }{T} & \;\;\;\;\;\;\;\;\;\;\;\;\;\;\;\;\;\;\;\;\;\;\;\;\;\;\;\;\;\;\;\;\;\;\;\;\;\;\;\;\;\;\;\;\;\;\;\;\;\;\;\;\;\;\;\;\;\;\;\;\mathrm{Ohm}’s\;\mathrm{law}\;\left(\mathrm{thermodynamic}\right) \end{array}$$

based on modified parameters $\alpha^{\prime }$ [J K m${}^{-1}$ s${}^{-1}$], $D_{c}^{\prime }$ [(mol species)${}^{2}$ K J${}^{-1}$ m${}^{-1}$ s${}^{-1}$] and ${D_{k}}^{\prime }$ [C${}^{2}$ K J${}^{-1}$ m${}^{-1}$ s${}^{-1}$], in which $\lambda =\kappa -\frac{2}{3}\mu$ is the second viscosity or first Lamé coefficient [Pa s], κ is the dilatational or volume viscosity [Pa s], δ is the Kronecker delta tensor and $\mu_{c}$ is the chemical potential of species *c* [J (mol species)${}^{-1}$]. In the second form of Newton’s law (32), ∇u=e+w is decomposed into strain rate and spin tensors $e=\frac{1}{2}\left(\nabla u+{\left(\nabla u\right)}^{⊤}\right)$ and $w=\frac{1}{2}\left(\nabla u-{\left(\nabla u\right)}^{⊤}\right)$ [65,88]. The second viscosity is not needed for incompressible flows (∇·u=0) or monatomic ideal gases (κ=0) [16,58,87].

We require simplified expressions for the above thermodynamic parameters. Comparing the equations for heat diffusion (24), (28) and (31) gives $\alpha^{\prime }=kT^{2}≃\alpha \rho c_{p}T^{2}$. For chemical diffusion, thermodynamic analysis (see Appendix B) yields $D_{c}^{\prime }≃\rho m_{c}D_{c}/R=p_{c}D_{c}/R^{2}T$ respectively for solutes or gaseous species, with strict equality for spatially invariant activity or fugacity coefficients, fluid density and temperature. Finally, comparing (27), (30) and (34) for Ohm’s law gives ${D_{k}}^{\prime }≃\kappa_{k}T≃z_{k}^{2}F^{2}C_{k}D_{k}/R=q_{k}^{2}n_{k}D_{k}/k_{B}$ for vanishing concentration and inverse temperature gradients.

#### 4.1.3. Entropy Production by Diffusion

For each of the above phenomena in isolation, the local unsteady entropy production (16) can be shown to reduce to [29,31,56,57,58,83,86,89]:

(35) $$\begin{array}{cc} {\hat{\dot{\sigma }}}_{\alpha }\; & =j_{Q}\cdot \nabla \frac{1}{T} \end{array}$$

(36) $$\begin{array}{cc} {\hat{\dot{\sigma }}}_{\nu }\; & =-\tau :\frac{\nabla u}{T} \end{array}$$

(37) $$\begin{array}{cc} {\hat{\dot{\sigma }}}_{D_{c}}\; & =-j_{c}\cdot \nabla \frac{\mu_{c}}{T} \end{array}$$

(38) $$\begin{array}{cc} {\hat{\dot{\sigma }}}_{D_{k}}\; & =-i_{k}\cdot \frac{\nabla \Phi }{T} \end{array}$$

where $a\cdot b=a^{⊤}b=\sum_{i}a_{i}b_{i}$ is the vector scalar product and $A:B=\mathrm{tr}\left(A^{⊤}B\right)=\sum_{i}\sum_{j}A_{ij}B_{ij}$ is the Frobenius tensor scalar product [90], used here for consistency with its norm. Substituting (31)–(34) and the above relations into (35)–(38) gives:

(39) $$\begin{array}{cc} {\hat{\dot{\sigma }}}_{\alpha }\; & ≃\alpha \rho c_{p}T^{2}\;{\left|\left|\nabla \frac{1}{T}\right|\right|}^{2}=\frac{1}{\alpha \rho c_{p}T^{2}}\;||j_{Q}{\left|\right|}^{2} \end{array}$$

(40) $$\begin{array}{c} {\hat{\dot{\sigma }}}_{\nu }\;=\frac{\rho \nu }{T} [{\left||\nabla u|\right|}^{2}+\mathrm{tr}\left({\left(\nabla u\right)}^{2}\right)]+\frac{\lambda }{T}\;{\left(\nabla \cdot u\right)}^{2}=\frac{1}{T} [2\mu e+\lambda \;\delta \;\mathrm{tr}(e)]:\nabla u\\ \;\;=\frac{1}{2\rho \nu T} [{\left||\tau |\right|}^{2}+\lambda \;\mathrm{tr}\left(\tau \right)\;\mathrm{tr}\left(e\right)]-\frac{\tau :w}{T} \end{array}$$

(41) $$\begin{array}{cc} {\hat{\dot{\sigma }}}_{D_{c}}\; & ≃\frac{\rho m_{c}D_{c}}{R}\;{\left|\left|\nabla \frac{\mu_{c}}{T}\right|\right|}^{2}=\frac{p_{c}D_{c}}{R^{2}T}\;{\left|\left|\nabla \frac{\mu_{c}}{T}\right|\right|}^{2}=\frac{R}{\rho m_{c}D_{c}}\;||j_{c}{\left|\right|}^{2}=\frac{R^{2}T}{p_{c}D_{c}}\;||j_{c}{\left|\right|}^{2} \end{array}$$

(42) $$\begin{array}{cc} {\hat{\dot{\sigma }}}_{D_{k}}\; & ≃\frac{z_{k}^{2}F^{2}C_{k}D_{k}}{RT^{2}}{\;||\nabla \Phi ||}^{2}=\frac{q_{k}^{2}n_{k}D_{k}}{k_{B}T^{2}}{\;||\nabla \Phi ||}^{2}=\frac{R}{z_{k}^{2}F^{2}C_{k}D_{k}}\;||i_{k}{\left|\right|}^{2}=\frac{k_{B}}{q_{k}^{2}n_{k}D_{k}}\;||i_{k}{\left|\right|}^{2} \end{array}$$

where $\left||a|\right|=\sqrt{a^{⊤}a}$ is the Euclidean norm for vector a, and $\left||A|\right|=\sqrt{\mathrm{tr}(A^{⊤}A)}$ is the Frobenius norm for tensor A. The first result or pair of results for each phenomenon in (39)–(42) apply to a fixed local gradient, thus to a *gradient-controlled system*, while the second result(s) apply to a fixed local flux, thus to a *flux-controlled system* [91].

#### 4.1.4. Entropic Similarity in Diffusion (Based on Entropy Production Terms)

We can now apply the principle of entropic similarity to construct dimensionless groups between individual diffusion processes and rank their relative importance. Consider the following ratios of local entropy production terms:

(43) $$\begin{array}{ccccccccc} {\hat{\Pi }}_{\nu /\alpha } & =\frac{{\hat{\dot{\sigma }}}_{\nu }}{{\hat{\dot{\sigma }}}_{\alpha }},\; & {\hat{\Pi }}_{\nu /D_{c}} & =\frac{{\hat{\dot{\sigma }}}_{\nu }}{{\hat{\dot{\sigma }}}_{D_{c}}},\; & {\hat{\Pi }}_{\nu /D_{k}} & =\frac{{\hat{\dot{\sigma }}}_{\nu }}{{\hat{\dot{\sigma }}}_{D_{k}}},\; & {\hat{\Pi }}_{\alpha /D_{c}} & =\frac{{\hat{\dot{\sigma }}}_{\alpha }}{{\hat{\dot{\sigma }}}_{D_{c}}},\; & \\ {\hat{\Pi }}_{\alpha /D_{k}} & =\frac{{\hat{\dot{\sigma }}}_{\alpha }}{{\hat{\dot{\sigma }}}_{D_{k}}},\; & {\hat{\Pi }}_{D_{c}/D_{k}} & =\frac{{\hat{\dot{\sigma }}}_{D_{c}}}{{\hat{\dot{\sigma }}}_{D_{k}}},\; & {\hat{\Pi }}_{D_{c}/D_{b}} & =\frac{{\hat{\dot{\sigma }}}_{D_{c}}}{{\hat{\dot{\sigma }}}_{D_{b}}},\; & {\hat{\Pi }}_{D_{k}/D_{\ell }} & =\frac{{\hat{\dot{\sigma }}}_{D_{k}}}{{\hat{\dot{\sigma }}}_{D_{\ell }}}\; \end{array}$$

where the second-last and final groups represent the relative effects of diffusion of chemical species *c* and *b*, and diffusion of charged species *k* and *ℓ*. Applying (39)–(42) under the assumption of constant gradients and various other properties, Equation (43) reduces to ratios of the corresponding diffusion coefficients:

(44) $$\begin{array}{ccccccc} {\hat{\Pi }}_{\nu /\alpha } & \to Pr=\frac{\nu }{\alpha }, & {\hat{\Pi }}_{\nu /D_{c}} & \to Sc_{c}=\frac{\nu }{D_{c}}, & {\hat{\Pi }}_{\nu /D_{k}} & \to Sc_{k}=\frac{\nu }{D_{k}}, & \\ {\hat{\Pi }}_{\alpha /D_{c}} & \to Le_{c}=\frac{\alpha }{D_{c}}, & {\hat{\Pi }}_{\alpha /D_{k}} & \to Le_{k}=\frac{\alpha }{D_{k}}, & {\hat{\Pi }}_{D_{c}/D_{k}} & \to \frac{D_{c}}{D_{k}}, & \\ {\hat{\Pi }}_{D_{c}/D_{b}} & \to \frac{D_{c}}{D_{b}}, & {\hat{\Pi }}_{D_{k}/D_{\ell }} & ≃\frac{z_{k}^{2}C_{k}D_{k}}{z_{\ell }^{2}C_{\ell }D_{\ell }}\to \frac{D_{k}}{D_{\ell }} \end{array}$$

These respectively give the Prandtl, Schmidt (species), Schmidt (charge), Lewis (species) and Lewis (charge) numbers (defined as the reciprocal in some references) and ratios of diffusion coefficients of chemical and/or charged species [15,16,58,77,79,80,92,93,94,95]. Alternatively, applying (39)–(42) for fixed fluxes rather than gradients, with other constant properties, (43) reduce to reciprocals of the groups in (44) and so, by convention, can also be represented by these groups. Additional groups can be defined for the second viscosity, e.g., from (40) for a flow-controlled system:

(45) $${\hat{\Pi }}_{\lambda /\mu }=\frac{\lambda {\left(\nabla \cdot u\right)}^{2}}{\mu [{\left||\nabla u|\right|}^{2}+\mathrm{tr}\left({\left(\nabla u\right)}^{2}\right)]}\to \frac{\lambda }{\mu }$$

Historically, the Prandtl, Schmidt and Lewis numbers are commonly derived by non-dimensionalization of the governing equations [16,92,93,94,95], by dimensional analysis [15,77] or directly by construction [16,58,77,79,80,93]. The principle of entropic similarity thus provides an alternative theoretical basis for these groups, which better reflects the underlying driving force—the maximization of entropy—for all diffusion phenomena.

It must be emphasized that the entropic groups based on (39)–(42) contain additional functional dependencies, for example on *T*, ρ, $c_{p}$, ∇·u, w, λ, $m_{c}$, $z_{k}$ and $C_{k}$ as well as the gradients and/or fluxes. Depending on the system, it may be necessary to preserve these parameters using the primary definitions of the entropic dimensionless groups given in (43). Furthermore, if one phenomenon is flux-controlled and the other is gradient-controlled, we obtain a hybrid dimensionless group, for example:

(46) $$\begin{array}{c} {\hat{\Pi }}_{\alpha /D_{c}}=\frac{\alpha T^{2}\rho^{2}c_{p}m_{c}D_{c}}{R}\frac{\;{\left|\left|\nabla T^{-1}\right|\right|}^{2}}{\left|\right|j_{c}{\left|\right|}^{2}} \end{array}$$

Such groups are not readily reducible to the ratios of diffusion coefficients (44) obtained by dimensional reasoning.

#### 4.1.5. Entropic Similarity in Diffusion (Based on Entropy Fluxes)

Also of interest for diffusion are the local non-fluid entropy fluxes, given respectively for independent flows of heat, chemical species and charged particles by the product of the flux and its corresponding intensive variable or field [29,31,56,57,58]:

(47) $$\begin{array}{c} j_{S,\alpha }=j_{Q}\frac{1}{T},\;j_{S,D_{c}}=-j_{c}\frac{\mu_{c}}{T},\;j_{S,D_{k}}=-i_{k}\frac{\Phi }{T} \end{array}$$

From the fluxes in (31), (33) and (34) these give, as functions of the gradients:

(48) $$\begin{array}{c} j_{S,\alpha }≃\alpha \rho c_{p}T\nabla \frac{1}{T},\;j_{S,D_{c}}≃\frac{\rho m_{c}D_{c}}{R}\frac{\mu_{c}}{T}\nabla \frac{\mu_{c}}{T},\;j_{S,D_{k}}≃\frac{z_{k}^{2}F^{2}C_{k}D_{k}}{R}\frac{\Phi }{T}\frac{\nabla \Phi }{T} \end{array}$$

While not dissipative, the fluid-borne entropy flux $j_{S,f}=\rho su$ in (12) is also important for entropy transport. The principle of entropic similarity can now be applied to assess the interplay between these entropy fluxes. First consider the flux magnitudes, which for fixed fluxes, gradients and other parameters give the following dimensionless groups:

(49) $$\begin{array}{c} \begin{array}{ccccc} {\hat{\Pi }}_{j_{S,\alpha }/j_{S,D_{c}}}\; & =\frac{\left|\right|j_{S,\alpha }\left|\right|}{\left|\right|j_{S,D_{c}}\left|\right|}\to Le_{c}=\frac{\alpha }{D_{c}},\; & {\hat{\Pi }}_{j_{S,\alpha }/j_{S,D_{k}}}\; & =\frac{\left|\right|j_{S,\alpha }\left|\right|}{\left|\right|j_{S,D_{k}}\left|\right|}\to Le_{k}=\frac{\alpha }{D_{k}},\; & \\ {\hat{\Pi }}_{j_{S,D_{c}}/j_{S,D_{k}}}\; & =\frac{\left|\right|j_{S,D_{c}}\left|\right|}{\left|\right|j_{S,D_{k}}\left|\right|}\to \frac{D_{c}}{D_{k}},\; & {\hat{\Pi }}_{j_{S,D_{c}}/j_{S,D_{b}}}\; & =\frac{\left|\right|j_{S,D_{c}}\left|\right|}{\left|\right|j_{S,D_{b}}\left|\right|}\to \frac{D_{c}}{D_{b}},\; & \\ {\hat{\Pi }}_{j_{S,D_{k}}/j_{S,D_{\ell }}}\; & =\frac{\left|\right|j_{S,D_{k}}\left|\right|}{\left|\right|j_{S,D_{\ell }}\left|\right|}≃\frac{z_{k}^{2}C_{k}D_{k}}{z_{\ell }^{2}C_{\ell }D_{\ell }}\to \frac{D_{k}}{D_{\ell }}\; & \\ {\hat{\Pi }}_{j_{S,\alpha }/j_{S,f}}\; & =\frac{\left|\right|j_{S,\alpha }\left|\right|}{\left|\right|j_{S,f}\left|\right|}≃\frac{\left|\right|j_{Q}\left|\right|}{\rho sT\;||u||}≃\frac{\alpha c_{p}T}{s}\frac{||\nabla T^{-1}\left|\right|}{\left||u|\right|},\\ {\hat{\Pi }}_{j_{S,D_{c}}/j_{S,f}}\; & =\frac{\left|\right|j_{S,D_{c}}\left|\right|}{\left|\right|j_{S,f}\left|\right|}≃\frac{\mu_{c}\;||j_{c}\left|\right|}{\rho sT\;||u||}≃\frac{D_{c}m_{c}\mu_{c}}{sRT}\frac{\left|\left|\nabla \frac{\mu_{c}}{T}\right|\right|}{\left||u|\right|},\\ {\hat{\Pi }}_{j_{S,D_{k}}/j_{S,f}}\; & =\frac{\left|\right|j_{S,D_{k}}\left|\right|}{\left|\right|j_{S,f}\left|\right|}≃\frac{\Phi \;||i_{k}\left|\right|}{\rho sT\;||u||}≃\frac{D_{k}z_{k}^{2}F^{2}C_{k}\Phi }{\rho sRT^{2}}\frac{\left||\nabla \Phi |\right|}{\left||u|\right|} \end{array} \end{array}$$

The first five groups in (49) reduce to the Lewis numbers and ratios of diffusion coefficients listed in (44). In contrast, the last three groups in (49) contain fluxes or gradients, the fluid velocity and other parameters and are not easily interpreted by kinematic or dynamic similarity. The relative importance of the fluid and non-fluid entropy fluxes can also be expressed by the composite group:

(50) $$\begin{array}{c} {\hat{\Pi }}_{j_{S,f}/j_{S}}=\frac{\left|\right|j_{S,f}\left|\right|}{\left|\right|j_{S}\left|\right|}=\frac{\left||\rho su|\right|}{\left|\right|j_{S,\alpha }+\sum_{c}j_{S,D_{c}}+\sum_{k}j_{S,D_{k}}\left|\right|} \end{array}$$

As previously noted, if the fluxes, gradients, fluid velocity or other parameters are not constant, it may be necessary to retain the unsimplified groups defined in (49) and (50).

The foregoing definitions in (49) and (50) discard the flux directions. A broader set of directional dimensionless groups can be constructed by reference to a unit normal n, e.g.:

(51) $$\begin{array}{cc} {\hat{\Pi }}_{j_{S,\alpha }/j_{S,D_{c}}}\left(n\right)\; & =\frac{j_{S,\alpha }\cdot n}{j_{S,D_{c}}\cdot n}, \end{array}$$

or alternatively by the use of normed dot products, e.g.:

(52) $$\begin{array}{cc} {\hat{\Pi }}_{j_{S,\alpha }/j_{S,D_{c}}}\; & =\frac{j_{S,\alpha }\cdot j_{S,D_{c}}}{\left|\right|j_{S,D_{c}}{\left|\right|}^{2}}, \end{array}$$

Equation (51) captures the directional dependence but will exhibit singularities associated with the direction (relative to n) of the denominator flux. Equation (52) provides a more robust dimensionless group, attaining a maximum when the component fluxes are equidirectional, decreasing to zero as the fluxes become orthogonal and decreasing further to a minimum (negative) value for antiparallel fluxes.

### 4.2. Chemical Reactions

#### 4.2.1. Thermodynamic Fundamentals

Although of different character, chemical reactions commonly occur in conjunction with one or more diffusion processes examined in Section 4.1. Continuous spontaneous chemical reactions also have non-zero entropy production, which can be expressed as the product of conjugate variables in an manner analogous to diffusion processes. For these reasons, we here examine the interplay between chemical reaction and diffusion processes from the perspective of entropic similarity.

The driving force for a chemical reaction is commonly represented by a *free energy*. A generalized dimensionless free energy concept, known as the generalized work or negative Massieu function ϝ, is obtained for a given set of constraints (in thermodynamics, a specific “ensemble”) by rearrangement of the maximum entropy relation (7) [23,24,26,27]:

(53) $$ϝ=-\ln Z=-\lambda_{0}=-H^{*}+\sum_{r=1}^{R}\lambda_{r}\langle f_{r}\rangle$$

It can be shown that spontaneous change occurs in the direction dϝ<0 [23,24,25,26,27,91]. For an ensemble defined by a constant temperature and volume, $k_{B}ϝ/T$ reduces to the Helmholtz free energy *F*, while for constant temperature and pressure, it gives the Gibbs free energy *G*. The latter (or the Planck potential −G/T, or a positive or negative affinity $A_{d}$) is most commonly used to analyze chemical reactions. Other free energy functions can be derived for different sets of constraints [20,89,91,96,97].

#### 4.2.2. Entropy Production by Chemical Reactions

For a continuous process, the local entropy production of the *d*th chemical reaction is given by [29,31,56,57,58,98]:

(54) $$\begin{array}{cc} {\hat{\dot{\sigma }}}_{d}\; & =-{\hat{\dot{\xi }}}_{d}\frac{\Delta {\tilde{G}}_{d}}{T} \end{array}$$

where $\Delta {\tilde{G}}_{d}$ is the change in molar Gibbs free energy for the *d*th reaction [J (mol reaction)${}^{-1}$], and ${\hat{\dot{\xi }}}_{d}$ is its rate of reaction per unit volume [(mol reaction) m${}^{-3}$ s${}^{-1}$]. To reduce (54), we express the change in Gibbs free energy as a function of chemical potentials in the reaction, then from (A1) in terms of chemical activities:

(55) $$\begin{array}{c} \Delta {\tilde{G}}_{d}=\sum_{c}\nu_{cd}\mu_{c}=\Delta {\tilde{G}}_{d}^{+}RT\ln\prod_{c}\alpha_{c}^{\nu_{cd}} \end{array}$$

where $\nu_{cd}$ is the stoichiometric coefficient of species *c* in the *d*th reaction [(mol species) (mol reaction)${}^{-1}$], with $\nu_{cd}>0$ for a product and $\nu_{cd}<0$ for a reactant, $\Delta {\tilde{G}}_{d}^{=}\sum_{c}\nu_{cd}\mu_{c}$ is the change in molar Gibbs free energy for the reaction under standard conditions, and $\prod_{c}\alpha_{c}^{\nu_{cd}}$ is termed the reaction quotient. The sum or product in (55) is taken over all species *c* participating in the reaction.

Some authors postulate a linear relation between the chemical reaction rate and driving force, analogous to Fick’s first law [98]. However, in contrast to diffusion, this assumption is not reasonable. Instead, the rate is generally expressed by the kinetic equation [78,80,99]:

(56) $$\begin{array}{c} {\hat{\dot{\xi }}}_{d}=k_{d}\prod_{c}{C_{c}}^{\beta_{cd}} \end{array}$$

where $k_{d}$ is the rate constant, $C_{c}$ is the molar concentration of chemical species *c* [(mol species) m${}^{-3}$] and $\beta_{cd}\in R$ is a power exponent [–] that must be found by experiment. A variety of concentration variables have been used in (56), including molar or mass concentrations, mole fractions, partial pressures or (rarely) activities or fugacities [80,99]. The units of $k_{d}$ depend on the concentration units and power exponents in (56). In general, the exponents $\beta_{cd}$ are unequal to the stoichiometric coefficients $\nu_{cd}$, but become equal for an elementary chemical reaction. For complex reactions, the total rate is the sum of rates for all individual steps or mechanisms with index *n*, such as forward and backward processes:

(57) $$\begin{array}{c} {\hat{\dot{\xi }}}_{d}=\sum_{n}{\hat{\dot{\xi }}}_{nd}=\sum_{n}k_{nd}\prod_{c}{C_{c}}^{\beta_{ncd}} \end{array}$$

Inserting (55) and (57) into (54) gives:

(58) $$\begin{array}{cc} {\hat{\dot{\sigma }}}_{d}\; & =-\left(\sum_{n}k_{nd}\prod_{c}{C_{c}}^{\beta_{ncd}}\right)\frac{\Delta {\tilde{G}}_{d}}{T}=-\left(\sum_{n}k_{nd}\prod_{c}{C_{c}}^{\beta_{ncd}}\right)\left(\frac{\Delta {\tilde{G}}_{d}}{T}+R\ln\prod_{c}\alpha_{c}^{\nu_{cd}}\right) \end{array}$$

The activities can be converted to molalities or partial pressures using (A1) and (A2), while the rate constants are often expressed in terms of activation energies by the Arrhenius equation [78,80,99].

#### 4.2.3. Entropic Similarity in Chemical Reactions, Diffusion and Fluid Transport

We now apply the principle of entropic similarity to construct entropic dimensionless groups between chemical reactions and transport phenomena:

(59) $$\begin{array}{cccccc} {\hat{\Pi }}_{d/e} & =\frac{{\hat{\dot{\sigma }}}_{d}}{{\hat{\dot{\sigma }}}_{e}},\; & {\hat{\Pi }}_{nd/md} & =\frac{{\hat{\dot{\sigma }}}_{nd}}{{\hat{\dot{\sigma }}}_{md}},\; & {\hat{\Pi }}_{d/\alpha } & =\frac{{\hat{\dot{\sigma }}}_{d}}{{\hat{\dot{\sigma }}}_{\alpha }},\;\\ {\hat{\Pi }}_{d/\nu } & =\frac{{\hat{\dot{\sigma }}}_{d}}{{\hat{\dot{\sigma }}}_{\nu }},\; & {\hat{\Pi }}_{d/D_{c}} & =\frac{{\hat{\dot{\sigma }}}_{d}}{{\hat{\dot{\sigma }}}_{D_{c}}},\; & {\hat{\Pi }}_{d/D_{k}} & =\frac{{\hat{\dot{\sigma }}}_{d}}{{\hat{\dot{\sigma }}}_{D_{k}}}\; \end{array}$$

The first two groups represent the competition between two single-mechanism chemical reactions *d* and *e*, or two mechanisms *n* and *m* for the same chemical reaction *d*. The remaining groups represent the entropic competition between the *d*th reaction and a diffusion process. Inserting (58) and the gradient forms of (39)–(42) into (59) gives:

(60) $$\begin{array}{c} \begin{array}{cc} {\hat{\Pi }}_{d/e}\; & =\frac{{\hat{\dot{\xi }}}_{d}\Delta {\tilde{G}}_{d}}{{\hat{\dot{\xi }}}_{e}\Delta {\tilde{G}}_{e}}=\frac{\left(k_{d}\prod_{c}{C_{c}}^{\beta_{cd}}\right)\Delta {\tilde{G}}_{d}}{\left(k_{e}\prod_{c}{C_{c}}^{\beta_{ce}}\right)\Delta {\tilde{G}}_{e}},\;{\hat{\Pi }}_{nd/md}=\frac{{\hat{\dot{\xi }}}_{nd}}{{\hat{\dot{\xi }}}_{md}}=\frac{k_{nd}\prod_{c}{C_{c}}^{\beta_{ncd}}}{k_{md}\prod_{c}{C_{c}}^{\beta_{mcd}}} \end{array} \end{array}$$

$$\begin{array}{ccc} {\hat{\Pi }}_{d/\alpha }\; & =\frac{{\hat{\dot{\xi }}}_{d}\Delta {\tilde{G}}_{d}}{{\hat{\dot{\sigma }}}_{\alpha }}=\frac{-\left(\sum_{n}k_{nd}\prod_{c}{C_{c}}^{\beta_{ncd}}\right)\Delta {\tilde{G}}_{d}}{\alpha \rho c_{p}T^{3}\;||\nabla T^{-1}{\left|\right|}^{2}}\; & ⇝\frac{k_{d}\;C_{c}^{\beta_{cd}}\;\Gamma_{\alpha d}}{\alpha },\\ {\hat{\Pi }}_{d/\nu }\; & =\frac{{\hat{\dot{\xi }}}_{d}\Delta {\tilde{G}}_{d}}{{\hat{\dot{\sigma }}}_{\nu }}=\frac{-\left(\sum_{n}k_{nd}\prod_{c}{C_{c}}^{\beta_{ncd}}\right)\Delta {\tilde{G}}_{d}}{{\rho \nu [||\nabla u||}^{2}+\mathrm{tr}\left({\left(\nabla u\right)}^{2}\right){]+\lambda \;\left(\nabla \cdot u\right)}^{2}}\; & ⇝\frac{k_{d}\;C_{c}^{\beta_{cd}}\;\Gamma_{\nu d}}{\nu },\\ {\hat{\Pi }}_{d/D_{c}}\; & =\frac{{\hat{\dot{\xi }}}_{d}\Delta {\tilde{G}}_{d}}{{\hat{\dot{\sigma }}}_{D_{c}}}=\frac{-R\left(\sum_{n}k_{nd}\prod_{c}{C_{c}}^{\beta_{ncd}}\right)\Delta {\tilde{G}}_{d}}{C_{c}D_{c}T{\left|\left|\nabla \frac{\mu_{c}}{T}\right|\right|}^{2}}\; & ⇝\frac{k_{d}\;C_{c}^{\beta_{cd}-1}\;\Gamma_{cd}}{D_{c}},\\ {\hat{\Pi }}_{d/D_{k}}\; & =\frac{{\hat{\dot{\xi }}}_{d}\Delta {\tilde{G}}_{d}}{{\hat{\dot{\sigma }}}_{D_{k}}}=\frac{-RT\left(\sum_{n}k_{nd}\prod_{c}{C_{c}}^{\beta_{ncd}}\right)\Delta {\tilde{G}}_{d}}{z_{k}^{2}F^{2}C_{k}{D_{k}||\nabla \Phi ||}^{2}}\; & ⇝\frac{k_{d}\;C_{c}^{\beta_{cd}}\;\Gamma_{kd}}{C_{k}D_{k}} \end{array}$$

where ⇝ indicates evaluation for a single mechanism with single-species kinetics, using the scaled free energy terms $\Gamma_{\alpha d}=-\Delta {\tilde{G}}_{d}/(\rho c_{p}T^{3}||\nabla T^{-1}{\left|\right|}^{2})$ [m${}^{5}$ mol${}^{-1}$], $\Gamma_{\nu d}≃-\Delta {\tilde{G}}_{d}/{\left(\rho [||\nabla u|\right|}^{2}+\mathrm{tr}\left({\left(\nabla u\right)}^{2}\right)])$ [m${}^{5}$ mol${}^{-1}$], $\Gamma_{cd}=-R\Delta {\tilde{G}}_{d}/(T||\nabla \left(\mu_{c}/T\right){\left|\right|}^{2})$ [m${}^{2}$] and $\Gamma_{kd}=-RT\Delta {\tilde{G}}_{d}/(z_{k}^{2}F^{2}$${\left||\nabla \Phi |\right|}^{2})$ [m${}^{2}$]. The last four groups can be interpreted as local modified Damköhler numbers, which compare the rate of entropy production by chemical reaction to that respectively from the diffusion of heat, momentum, chemical species or charge [58,80].

For fixed fluxes rather than gradients, the flux terms in (39)–(42) yield a different set of groups, e.g.:

(61) $${\hat{\Pi }}_{d/D_{c}}=\frac{-\left(\sum_{n}k_{nd}\prod_{c}{C_{c}}^{\beta_{ncd}}\right)\Delta {\tilde{G}}_{d}C_{c}D_{c}}{RT||j_{c}{\left|\right|}^{2}}⇝\frac{-\left(k_{d}{C_{c}}^{\beta_{cd}+1}\right)\Delta {\tilde{G}}_{d}D_{c}}{RT||j_{c}{\left|\right|}^{2}}$$

Hybrid groups based on other assumptions [80] are also possible.

Entropic dimensionless groups can also be constructed by comparing the entropy production by chemical reaction (58) to the entropy flux of a diffusion or fluid transport process (48), by inclusion of a length scale *ℓ* [m]. For chemical reaction relative to the diffusion of heat, chemical species or charge, this yields groups containing the normalized gradient $\ell^{-1}∼\left||\nabla X|\right|/X$ analogous to those in (60), where *X* is the intensive variable or field. For chemical reaction relative to fluid transport, we obtain:

(62) $$\begin{array}{cc} {\hat{\Pi }}_{{\hat{\dot{\sigma }}}_{d}/j_{S,f}}\; & =\frac{{\hat{\dot{\sigma }}}_{d}\ell }{\left|\right|j_{S,f}\left|\right|}=\frac{-\left(\sum_{n}k_{nd}\prod_{c}{C_{c}}^{\beta_{ncd}}\right)\Delta {\tilde{G}}_{d}\ell }{\rho sT||u||} \end{array}$$

The latter has no equivalent in (60).

For chemical reactions in which the free energy $\Delta {\tilde{G}}_{d}$ can be considered constant—such as for steady-state conditions—the free energy component of the entropy production (54) can be disregarded, enabling the formation of entropic groups based solely on the reaction rate ${\hat{\dot{\xi }}}_{d}$. Comparing chemical reactions *d* and *e*, or reaction mechanisms *m* and *n*, by entropic similarity gives the following groups (compare (60)):

(63) $$\begin{array}{cc} {\hat{\Pi }}_{d/e}\; & =\frac{{\hat{\dot{\xi }}}_{d}}{{\hat{\dot{\xi }}}_{e}}=\frac{k_{d}\prod_{c}{C_{c}}^{\beta_{cd}}}{k_{e}\prod_{c}{C_{c}}^{\beta_{ce}}},\;{\hat{\Pi }}_{nd/md}=\frac{{\hat{\dot{\xi }}}_{nd}}{{\hat{\dot{\xi }}}_{md}}=\frac{k_{nd}\prod_{c}{C_{c}}^{\beta_{ncd}}}{k_{md}\prod_{c}{C_{c}}^{\beta_{mcd}}} \end{array}$$

For process engineering applications, the reaction rate can also be compared to fluid transport processes by an extended Damköhler number [58,80]:

(64) $$\begin{array}{cc} {\hat{\Pi }}_{d/\theta }=\frac{{\hat{\dot{\xi }}}_{d}\theta }{\prod_{c}C_{c}}=\left(\sum_{n}k_{nd}\prod_{c}{C_{c}}^{\beta_{ncd}-1}\right)\theta \; & ⇝Da_{d}=k_{d}\;C_{c}^{\beta_{cd}-1}\theta \end{array}$$

where θ is a transport or residence time scale [s]. For a single mechanism and single-species kinetics, this reduces to the standard Damköhler number. Examples of the residence time include θ=V/Q for a continuously mixed flow reactor, where *V* is its volume [m${}^{3}$] and *Q* is the volumetric flow rate [m${}^{3}$ s${}^{-1}$], or θ=L/U for a plug-flow reactor, where *L* is its length [m] and *U* is the superficial velocity [m s${}^{-1}$] [58,80].

### 4.3. Diffusion and Chemical Reaction Cross-Phenomena

#### 4.3.1. Description and Onsager Relations

In thermodynamic systems, it is necessary to consider the occurrence of diffusion and chemical reaction cross-phenomena, in which a thermodynamic force conjugate to one quantity induces the flow of a different quantity or vice versa. A number of pairwise interactions—based on the previously examined phenomena and also pressure gradients and magnetic fields—are listed in Table 1. Some ternary interactions (e.g., thermomagnetic convection, Nernst effect and Ettinghausen effect) are also known [77].

For small gradients (or small “distance from equilibrium”), the pairwise interactions are commonly represented by the Onsager relations [100,101], derived from the assumption of microscopic reversibility [31,56,77,89,98,102,103,104]:

(65) $$\begin{array}{c} j_{rıj}=\sum_{m\kappa \ell }L_{rıj,m\kappa \ell }\;f_{m\kappa \ell } \end{array}$$

where $j_{rıj}$$\in \{j_{Q},\tau ,\left\{j_{c}\right\},\left\{i_{k}\right\},\left\{{\hat{\dot{\xi }}}_{d}\right\}\}$ is the ıjth component of the *r*th flux or rate, $f_{m\kappa \ell }$$\in \left\{\nabla T^{-1},-\nabla u/T,\left\{-\nabla \left(\mu_{c}/T\right)\right\},-\nabla \Phi /T,\left\{-\Delta {\tilde{G}}_{d}/T\right\}\right\}$ is the κℓth component of the *m*th thermodynamic force and $L_{rıj,m\kappa \ell }$ is the phenomenological coefficient or generalized conductance, where ı,j,κ,ℓ∈{x,y,z} are Cartesian coordinate components with j,ℓ redundant for vectors and all indices redundant for scalars. By reindexing the components $j_{rıj}\mapsto j_{a}$, $f_{m\kappa \ell }\mapsto f_{b}$ and $L_{rıj,m\kappa \ell }\mapsto L_{a,b}$, Equation (65) can be assembled into the vector-tensor form:

(66) $$\begin{array}{c} j=L\;f \end{array}$$

where j is the flux vector, f is the thermodynamic force vector and L is a two-dimensional matrix of phenomenological coefficients.

Many authors restrict (65) and (66) using the “Curie principle” [105], commonly interpreted to allow coupling only between phenomena of the same tensorial order [56,77,98]. Under this principle, the velocity gradient ∇u, a second-order tensor, is unable to influence the fluxes of heat $j_{Q}$ or chemical species $j_{c}$, these being first-order tensors (vectors). In turn, the fluxes are unable to influence the scalar chemical reaction rates ${\hat{\dot{\xi }}}_{d}$. However, the velocity divergence ∇·u, a scalar, is able to influence the scalar reaction rates ${\hat{\dot{\xi }}}_{d}$. The Curie principle can be derived under the linear assumption (65) and the invariance of an isotropic tensor to coordinate inversion [56,98]. However, due to continued controversy over its definition and validity [106,107,108,109,110,111] and deeper connections between tensorial order and group theory [112,113,114,115], this study adopts the most general formulation. We also recall the concerns expressed in Section 4.2 on the validity of the linear assumption for chemical reactions, hence these components of (65) and (66) may have limited applicability.

**Table 1:** Reported examples of thermodynamic cross-phenomena, showing pairwise couplings between thermodynamic forces and fluxes [28,31,77,89,98,100,101,102,115].

**Phenomenon**	Fluid flux	Heat flux	Momentum flux (viscous stress tensor)	Chemical flux	Charge flux	Magnetic flux	Chemical reaction rate
Pressure gradient	Fluid flow (Poiseuille)	Fluid flow (dissipative); Forced convection	Turbulent flow	Reverse osmosis	Streaming current	Magnetic pressure	Chemical–mechanical coupling
Temperature gradient	Thermal transpiration; Thermal osmosis; Free convection	Thermal conduction		Thermo-diffusion/ Soret effect	Thermo-electric (Seebeck) effect; Thomson effect	Thermo-magnetism	Reaction-induced temperature gradient
Velocity gradient	Fluid flow (Couette)		Momentum diffusion	Tollert effect			
Chemical potential gradient	Osmosis	Dufour effect; Latent heat transfer		Chemical diffusion; Co-diffusion	Electro-chemical transference; Donnan effect		Reaction-induced chemical gradient
Electric field	Electro-osmosis	Thermo-electric (Peltier) effect	Electro-viscous coupling	Hittorf transference	Charge diffusion (drift)	Electro-magnetism	Electrolytic cell
Magnetic field	Magneto-osmosis; Magneto-hydrodynamic effect	Thermo-magnetism	Magneto-viscous coupling		Electro-magnetic induction	Magnetism	
Free energy difference	Reaction osmosis	Reaction-induced heat flux		Reaction-induced chemical flux	Electro-chemical (galvanic) cell		Chemical reaction; Coupled reactions

#### 4.3.2. Entropy Production and Entropic Similarity

Incorporating diffusion and chemical reaction cross-phenomena, the total local entropy production is:

(67) $$\begin{array}{c} {\hat{\dot{\sigma }}}_{\mathrm{tot}}=j\cdot f={\left(L\;f\right)}^{⊤}f=f^{⊤}L^{⊤}f=j^{⊤}L^{-1}j\geq 0 \end{array}$$

This subsumes diffusion and reaction processes acting in isolation, (35)–(38) and (54). Equation (67) also allows some individual phenomena to decrease the entropy production, provided that the total is non-negative.

Applying the principle of entropic similarity, the total local entropy production (67) can be scaled by any reference entropy production term, giving the dimensionless group:

(68) $$\begin{array}{c} {\hat{\Pi }}_{\mathrm{tot}}=\frac{{\hat{\dot{\sigma }}}_{\mathrm{tot}}}{{\hat{\dot{\sigma }}}_{\mathrm{ref}}}=\frac{f^{⊤}L^{⊤}f}{j_{\mathrm{ref}}\cdot f_{\mathrm{ref}}} \end{array}$$

Generally, it is necessary to handle a mix of different units in j, f and L. In some cases, j and f can be chosen so that all phenomenological coefficients $L_{a,b}\in L$ have consistent units such as [m${}^{2}$ s${}^{-1}$]. By entropic similarity, an extended family of groups can also be derived for competing cross-phenomenological processes, analogous to those in (43) and (44):

(69) $$\begin{array}{c} \begin{array}{c} {\hat{\Pi }}_{L_{c,d}/L_{a,b}}=\frac{{\hat{\dot{\sigma }}}_{L_{c,d}}}{{\hat{\dot{\sigma }}}_{L_{a,b}}}\to \frac{L_{c,d}}{L_{a,b}} \end{array} \end{array}$$

Equation (69) assumes constant gradients and other properties and common units for $L_{ı,j}$.

## 5. Dispersion Processes

We now consider *dispersion* processes, consisting of mixing or spreading phenomena analogous to diffusion but arising from processes at microscopic to macroscopic rather than molecular scales. Commonly, these are represented by dispersion coefficients of the same dimensions [m${}^{2}$ s${}^{-1}$] as practical diffusion coefficients. A number of dispersion processes are discussed in turn.

### 5.1. Inertial Dispersion

**Figure 1:**
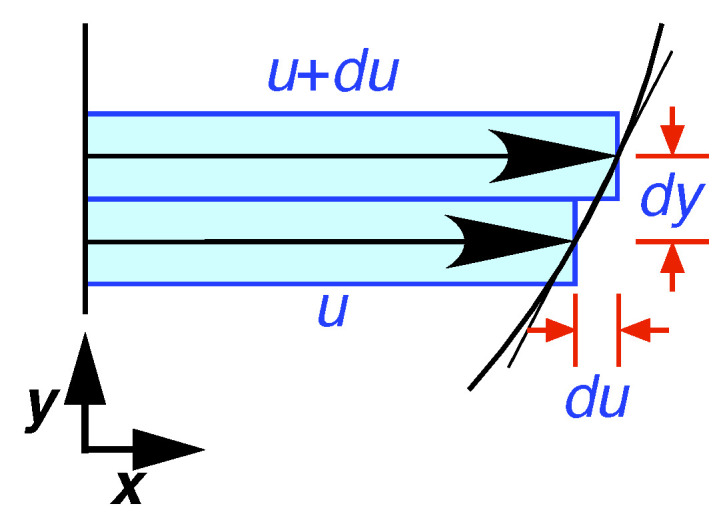
Differential motion of parallel fluid elements and the resulting orthogonal velocity gradient.

In fluid flow, the foremost dispersion process—here termed *inertial dispersion*—involves the spreading of momentum and any fluid-borne properties (e.g., heat, chemical species, charge) due to inertial flow. This can be demonstrated by the classical diagram of inertial effects in a fluid flow, given in Figure 1 [13,103]. As shown, the velocity difference between two adjacent fluid elements moving in (say) the *x*-direction will create a velocity gradient normal to the flow, here drawn in the *y* direction. Above a threshold, the resulting shear stresses will produce inertial motions of fluid normal and opposite to the velocity gradient, causing the lateral transfer of momentum from regions of high to low momentum. Similarly, a velocity gradient in the flow direction, say *x*, will tend to be counterbalanced by opposing inertial flows. Taken together, these effects cause the transfer of momentum in opposition to the velocity gradient tensor, producing a flow field which is more spatially uniform in the mean but with a tendency toward turbulent flow.

#### 5.1.1. Entropic Similarity for Inertial Dispersion in Internal Flows

First consider *internal flows*, involving flow in a conduit with solid walls under a pressure gradient (Poiseuille flow). For steady irrotational incompressible flow in a cylindrical pipe, the total entropy production is [30,76,116,117,118]:

(70) $$\begin{array}{c} {\dot{\sigma }}_{\mathrm{int}}=\frac{p_{L}Q}{T}=\frac{\rho gH_{L}Q}{T}=\frac{\pi \rho gH_{L}Ud^{2}}{4T} \end{array}$$

where $p_{L}$ is the pressure loss [Pa], $H_{L}$ is the head loss [m], *Q* is volumetric flow rate [m${}^{3}$ s${}^{-1}$], *U* is the mean velocity [m s${}^{-1}$] and *d* is the pipe diameter [m]. The head loss by inertial flow is given by the Darcy–Weisbach equation [11,13,15,16,17,18,88]:

(71) $$\begin{array}{c} H_{L,I}=\frac{fL}{2gd}U^{2} \end{array}$$

where *f* is the Darcy friction factor [–] for the inertial regime and *L* is the pipe length [m]. Substituting in (70) gives the entropy production by inertial dispersion:

(72) $$\begin{array}{c} {\dot{\sigma }}_{\mathrm{int},I}=\frac{\pi \rho dfL}{8T}U^{3} \end{array}$$

Equations (70)–(72) allow for flow reversal, with $p_{L}<0$, $H_{L}<0$ and f<0 corresponding to U<0 and Q<0, giving ${\dot{\sigma }}_{\mathrm{int},I}\geq 0$ in all cases. For laminar flow involving purely viscous diffusion, the head loss and entropy production are, analytically [11,13,93]:

(73) $$\begin{array}{c} H_{L,\nu }=\frac{32\nu L}{gd^{2}}U,\;{\dot{\sigma }}_{\mathrm{int},\nu }=\frac{8\pi \rho \nu L}{T}U^{2}. \end{array}$$

The relative importance of inertial dispersion and viscous diffusion for an internal flow can therefore be examined by the entropic group:

(74) $$\begin{array}{c} \Pi_{\mathrm{int},I/\nu }=\frac{{\dot{\sigma }}_{\mathrm{int},I}}{{\dot{\sigma }}_{\mathrm{int},\nu }}=\frac{f\;Ud}{64\nu }=\frac{fRe}{64}∼fRe,\;\mathrm{with}\;Re=\frac{Ud}{\nu } \end{array}$$

where Re is the Reynolds number, normally derived by dynamic similarity (1). The group Po=fRe/2 has been termed the Poiseuille number [16,119]. For purely viscous diffusion (laminar flow), f=64/Re [11,13] and $\Pi_{\mathrm{int},I/\nu }=1$. For inertial flows $\Pi_{\mathrm{int},I/\nu }>1$, *f* is commonly correlated with Re and ϵ/d, where ϵ is the equivalent sand roughness of the pipe [m] [120]. For non-circular conduits, *d* is replaced by the hydraulic diameter $d_{H}=4A/P_{w}$, where *A* is the area [m${}^{2}$] and $P_{w}$ is the wetted perimeter [m] [16,88,93]. For wall shear flows, Equation (74) is written using the friction velocity $u^{*}=\sqrt{\tau_{0}/\rho }=U\sqrt{f/8}$, where $\tau_{0}$ is the wall shear stress [Pa], giving $\Pi_{\mathrm{int},I/\nu }∼\sqrt{f}\;Re^{*}$ with $Re^{*}=u^{*}d/\nu$ [13]. For flow in porous media, the pressure loss is given by the Ergun equation [121], within which replacing *d* by a void length scale and *U* by an interstitial velocity gives a more natural representation [119,122].

Interpreting (74) from the perspective of entropic similarity, within an internal flow the velocity will decrease from the order of its mean velocity *U* to zero over distances of the order of its length scale *d* (see Figure 1). Above a critical threshold, these will produce inertial flows with a macroscopic dispersion coefficient of order Ud. In consequence, the Reynolds number can be interpreted, from an entropic perspective, simply as the ratio of the inertial dispersion and viscous diffusion coefficients. Multiplication by *f* to give the entropic group $\Pi_{\mathrm{int},I/\nu }$ incorporates the resistance of the conduit to the imposed flow, including the effects of flow regime, pipe geometry and surface roughness.

**Figure 2:**
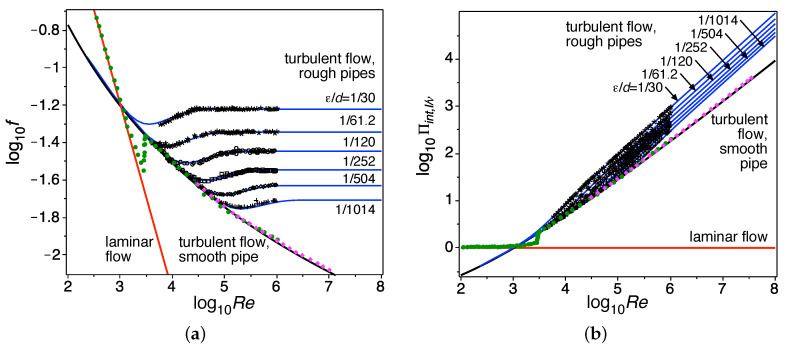
Entropic similarity diagrams for internal flow in a cylindrical pipe: (**a**) *f* versus Re (the Moody [123] diagram) and (**b**) $\Pi_{\mathrm{int},I/\nu }$ versus Re. These include experimental data points for rough pipes (black symbols) [124] and smooth pipes (green and magenta symbols) [125], the laminar flow curve f=64/Re (red) and fitted curves for turbulent flow in a smooth pipe $f^{-1/2}=2\log_{10}\left(Re\sqrt{f}\right)-0.795$ (black) [120] and in rough pipes $f^{-1/2}=1.128+0.64\left(0.96^{k_{s}^{+}}\right)-2.62\left(0.64^{k_{s}^{+}}\right)-2\log_{10}\left(\epsilon /d\right)$ (blue), where $k_{s}^{+}=u^{*}\epsilon /\nu =Re\;\left(\epsilon /d\right)\sqrt{f/8}$ [126].

For internal flows, the characteristic plot of *f* against Re (the Moody [123] diagram) is shown in Figure 2a, showing theoretical laminar and fitted turbulent curves for flow in smooth and rough pipes, with supporting experimental data [124,125,126]. This can be interpreted as an *entropic similarity diagram*, showing the dominant entropic regimes for different flow conditions. This information can be converted to a plot of $\Pi_{\mathrm{int},I/\nu }$ (74) against Re, shown in Figure 2b [118]. This plot provides a clear separation between the occurrence of laminar flow $\Pi_{\mathrm{int},I/\nu }=1$ at low Re, and the transition to turbulent flow $\Pi_{\mathrm{int},I/\nu }>1$ at higher Re, with different curves for smooth pipes or pipes of different roughness. Examining the experimental data shown [125], the laminar region is metastable beyond the predicted transition at Re=1038, with a sharp transition occurring at Re∼2800. This plot demonstrates the value of the entropic group $\Pi_{\mathrm{int},I/\nu }$ for the interpretation of flow regimes in internal flows.

#### 5.1.2. Entropic Similarity for Inertial Dispersion in External Flows

Now consider *external flows*, involving flow around a solid object [11,13,16,17,18]. For a simplified steady one-dimensional flow, the entropy production is [30,76,116,117]:

(75) $$\begin{array}{c} {\dot{\sigma }}_{\mathrm{ext}}=\frac{F_{D}U}{T} \end{array}$$

where $F_{D}$ is the magnitude of the drag force [N] and *U* is the mean velocity of the ambient fluid [m s${}^{-1}$]. The drag force is commonly scaled by the dynamic pressure to give a dimensionless drag coefficient [11,13,93]:

(76) $$\begin{array}{c} C_{D}=\frac{F_{D}}{\frac{1}{2}\rho A_{s}U^{2}} \end{array}$$

where $A_{s}$ is the cross-sectional area of the solid normal to the flow [m${}^{2}$]. Taking $A_{s}=\pi d^{2}/4$ for a sphere, where *d* is the diameter [m], the inertial entropy production is:

(77) $$\begin{array}{c} {\dot{\sigma }}_{\mathrm{ext},I}=\frac{\rho A_{s}C_{D}U^{3}}{2T}=\frac{\pi \rho d^{2}C_{D}U^{3}}{8T} \end{array}$$

Equations (75)–(77) allow for flow reversal, with $F_{D}<0$ and $C_{D}<0$ corresponding to U<0, for which ${\dot{\sigma }}_{\mathrm{ext},I}\geq 0$ in all cases. For steady laminar (Stokes) flow around a sphere, the drag force and entropy production are, analytically [11,13,16,93]:

(78) $$\begin{array}{c} F_{D}=3\pi \rho \nu dU,\;{\dot{\sigma }}_{\mathrm{ext},\nu }=\frac{3\pi \rho \nu dU^{2}}{T} \end{array}$$

The relative importance of inertial dispersion and viscous diffusion for an external flow can therefore be assessed by the entropic group [127]:

(79) $$\begin{array}{c} \Pi_{\mathrm{ext},I/\nu }=\frac{{\dot{\sigma }}_{\mathrm{ext},I}}{{\dot{\sigma }}_{\mathrm{ext},\nu }}=\frac{C_{D}\;Ud}{24\nu }=\frac{C_{D}Re}{24}∼C_{D}Re,\;\mathrm{with}\;Re=\frac{Ud}{\nu } \end{array}$$

This is analogous to (74), with an equivalent entropic interpretation. For non-spherical solids, $A_{s}$ can be used directly, or *d* assigned to a representative length scale for the solid. For purely viscous diffusion, $C_{D}=24/Re$ [11,13,93] and $\Pi_{\mathrm{ext},I/\nu }=1$. For inertial flows $\Pi_{\mathrm{ext},I/\nu }>1$, many correlations of $C_{D}$ with Re have been formulated for application to a wide variety of solid shapes [63,128,129,130,131].

**Figure 3:**
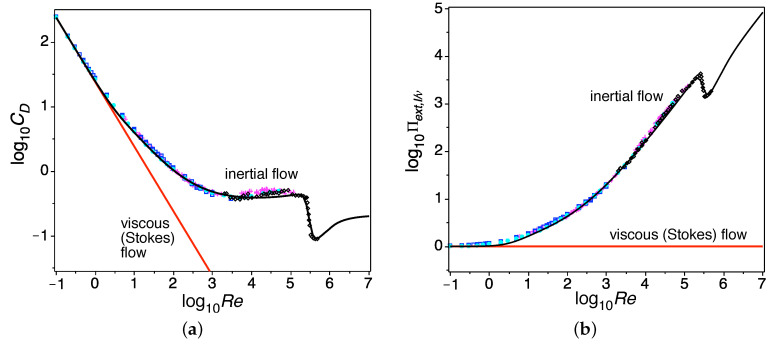
Entropic similarity diagrams for external flow around a sphere: (**a**) $C_{D}$ versus Re and (**b**) $\Pi_{\mathrm{ext},I/\nu }$ versus Re. These include experimental data points [93] (black symbols), [132] (magenta), [133] (cyan) and [134] (blue), the viscous Stokes flow curve $C_{D}=24/Re$ (red), and the Morrison correlation $C_{D}\left(Re\right)$ for $Re\leq 10^{6}$ [130] (black) with $C_{D}=0.21-8\times 10^{4}/Re$ for $Re>10^{6}$ (compare [128]).

For external flows, the characteristic plot of $C_{D}$ against Re is shown in Figure 3a, showing theoretical viscous and fitted inertial flow curves, with supporting experimental data [93,130,132,133,134]. This is also an *entropic similarity diagram*, showing the dominant entropic regimes. It can further be converted to a plot of $\Pi_{\mathrm{ext},I/\nu }$ (79) against Re, shown in Figure 3b [127]. This plot provides a clear separation between the occurrence of viscous flow $\Pi_{\mathrm{ext},I/\nu }=1$ at low Re, and its gradual transition to inertial flow $\Pi_{\mathrm{ext},I/\nu }>1$ at higher Re. As also shown, the “drag crisis”—the sharp reduction in $C_{D}$ at high Reynolds numbers due to the collapse of the laminar boundary layer—is associated with a dramatic drop in the entropy production. This plot demonstrates the utility of $\Pi_{\mathrm{ext},I/\nu }$ for the interpretation of flow regimes in external flows.

The above treatment can be extended to more complicated flows. For two- or three-dimensional steady irrotational external flows, it is necessary to consider the drag force and lift force(s) respectively aligned with and normal to a reference direction, each with a corresponding drag or lift coefficient, for which a vector formulation is useful. Consider a solid object moving at velocity V [m s${}^{-1}$] in a uniform flow field of ambient velocity u [m s${}^{-1}$], producing the vector drag–lift force $F_{D}$ [N] on the object. The vector drag–lift coefficient $C_{D}={\left[C_{D},C_{L}\right]}^{⊤}$ or ${\left[C_{D},C_{L1},C_{L2}\right]}^{⊤}$ and inertial entropy production are [76]:

(80) $$\begin{array}{c} C_{D}=\frac{F_{D}}{\frac{1}{2}\rho A_{s}||u-{V||}^{2}} \end{array}$$

(81) $$\begin{array}{c} {\dot{\sigma }}_{\mathrm{ext},I}=\frac{F_{D}\cdot \left(u-V\right)}{T}=\frac{\frac{1}{2}\rho A_{s}||u-{V||}^{2}\;C_{D}\cdot \left(u-V\right)}{T} \end{array}$$

while the Stokes viscous force and viscous entropy production for a sphere are:

(82) $$\begin{array}{c} F_{D}=3\pi \rho \nu d\left(u-V\right)\;\mathrm{and}\;{\dot{\sigma }}_{\mathrm{ext},\nu }=\frac{3\pi \rho \nu d\;||u-{V||}^{2}}{T} \end{array}$$

The entropic dimensionless group (79), taking $A_{s}=\pi d^{2}/4$, becomes:

(83) Πext,I/ν=σ˙ext,Iσ˙ext,ν=CD·(u−V)d24ν∼CD·RewithRe=(u−V)dν

where Re is a vector Reynolds number, which accounts for the ratio of the inertial dispersion and viscous diffusion coefficients in each direction. The drag and lift directions are quite distinct, since lift forces are unrelated to viscous friction [135] but are governed by the fluid circulation $\Gamma =-\oint_{C}u\cdot ds$ on any closed path C around the solid, where s is the path coordinate [11,13,18,88].

Now consider the pure rotation of a rigid sphere of radius vector r [m] about its centroid $x_{G}$ at the angular velocity ω(t) [s${}^{-1}$]. The entropy production is:

(84) $$\begin{array}{c} {\dot{\sigma }}_{\mathrm{ext}}^{\mathrm{rot}}=\frac{T\cdot \omega }{T} \end{array}$$

where T is the torque on the solid [N m]. The viscous torque on the sphere is $T={8\pi \mu ||r||}^{3}\omega$ [135,136,137]. The ratio of rotational inertial to viscous effects can therefore be represented by the entropic group:

(85) Πext,I/νrot=σ˙ext,Irotσ˙ext,νrot=CT·ReT16π∼CT·ReTwithReT=||r||2ων,CT=T12ρ||r||5||ω||2

where ReT is a rotational Reynolds (or Taylor) number and $C_{T}$ is a torque coefficient, in which ReT and $C_{T}$ are pseudovectors [93,137,138]. Clearly, ReT can be interpreted as the ratio of the rotational inertial dispersion coefficient ${\left||r|\right|}^{2}\omega$ to the viscous diffusion coefficient ν, while its multiplication by $C_{T}$ incorporates the resistance to rotation. For purely viscous diffusion, CT=16πReT/||ReT||2 [137] and $\Pi_{\mathrm{ext},I/\nu }^{\mathrm{rot}}=1$. Equation (85) can be extended to different solid shapes and centers of rotation based on moments of inertia.

For unsteady irrotational external flows, it is necessary to consider the inertial drag due to the “added mass” of fluid, a history-dependent force and the acceleration of the local fluid [139,140,141]. For combined translational and rotational flows, it is necessary to consider the Magnus force due to rotation-induced lift [135,138], with additional contributions from flow-induced or imposed vibrations [142] and deformable solids such as flapping wings.

For boundary layer flows such as along a flat plate, it is usual to consider a local drag coefficient $C_{D}\left(x\right)$ and boundary layer thickness δ(x) as functions of *x* [13,93]. These give the local dimensionless groups $\Pi_{I,x/\nu }\left(x\right)=C_{D}\left(x\right)Re_{x}\left(x\right)$ and $\Pi_{I,\delta /\nu }\left(x\right)=C_{D}\left(x\right)Re_{\delta }\left(x\right)$, where $Re_{x}\left(x\right)=U_{\infty }x/\nu$, $Re_{\delta }\left(x\right)=U_{\infty }\delta \left(x\right)/\nu$ and $U_{\infty }$ is the free-stream velocity. The Reynolds numbers assess the importance of local inertial dispersion, measured by $U_{\infty }x$ or $U_{\infty }\delta \left(x\right)$, relative to viscous diffusion.

#### 5.1.3. Entropic Similarity for Inertial Dispersion with Other Diffusion Processes

Since inertial dispersion dramatically enhances the spreading of other fluid properties, it is necessary to consider its influence relative to other diffusion processes. Drawing an analogy between macroscopic heat, chemical species or ionic diffusion and laminar viscous diffusion in an internal flow (73), this can be represented by the entropic groups [14,58,77,80,93,128]:

(86) $$\begin{array}{c} \begin{array}{ccc} \Pi_{\mathrm{int},I/\alpha }=\frac{{\dot{\sigma }}_{\mathrm{int},I}}{{\dot{\sigma }}_{\mathrm{int},\alpha }}∼fPe_{\alpha },\; & \; & \mathrm{with}\;Pe_{\alpha }=\frac{Ud}{\alpha }=RePr,\\ \Pi_{\mathrm{int},I/c}=\frac{{\dot{\sigma }}_{\mathrm{int},I}}{{\dot{\sigma }}_{\mathrm{int},D_{c}}}∼fPe_{c},\; & \; & \mathrm{with}\;Pe_{c}=\frac{Ud}{D_{c}}=ReSc_{c},\\ \Pi_{\mathrm{int},I/k}=\frac{{\dot{\sigma }}_{\mathrm{int},I}}{{\dot{\sigma }}_{\mathrm{int},D_{k}}}∼fPe_{k},\; & \; & \mathrm{with}\;Pe_{k}=\frac{Ud}{D_{k}}=ReSc_{k} \end{array} \end{array}$$

where *d* is a length scale [m], and $Pe_{\alpha },Pe_{c}$ and $Pe_{k}$ are Péclet numbers respectively for heat, chemical species *c* or charged species *k*. Each Péclet number is the ratio of the inertial dispersion coefficient Ud to the heat, chemical species or charge diffusion coefficient, equivalent to the product of the Reynolds number (74) and the corresponding Prandtl or Schmidt number (44). In process engineering, the length scales in (86) are substituted by a length scale *L* of the reactor [80].

### 5.2. Turbulent Dispersion

An important mixing process in fluid flow is *turbulent dispersion*, often termed *eddy dispersion* or *eddy diffusion*, caused by turbulent motions of the fluid. This provides the dominant mixing mechanism for many natural flow systems, including flows in streams, lakes, oceans and atmosphere [15]. Turbulent dispersion is closely related to the inertial dispersion but is analyzed at local scales using the Reynolds decomposition of each physical quantity $a=\bar{a}+a^{\prime }$, where $\bar{a}$ is a Reynolds average (such as the time or ensemble average) and $a^{\prime }$ is the fluctuating component [14,65,93]. As shown by Reynolds [143], averaging of the incompressible Navier–Stokes equations reveals an additional contribution to the mean stress tensor $\bar{\tau_{t}}=\rho \bar{u^{\prime }u^{\prime }}$ [Pa], known as the Reynolds stress tensor. This is often correlated empirically by the Boussinesq approximation [65,144,145]:

(87) $$\begin{array}{c} \bar{\tau_{t}}=\rho \bar{u^{\prime }u^{\prime }}\approx -\rho \nu_{t}\left(\nabla \bar{u}+{\left(\nabla \bar{u}\right)}^{⊤}\right)+\frac{1}{3}\rho \delta \;\mathrm{tr}\left(\bar{u^{\prime }u^{\prime }}\right) \end{array}$$

where $\nu_{t}$ is the turbulent dispersion coefficient or eddy viscosity [m${}^{2}$ s${}^{-1}$]. Similarly, the Reynolds average of conservation laws for heat, chemical species and charge reveals mean fluctuating (Reynolds) fluxes, commonly correlated by:

(88) $$\begin{array}{c} \begin{array}{cccc} \bar{j_{Q,t}}\; & =\bar{u^{\prime }{\left(\rho c_{p}T\right)}^{\prime }}\; & \; & \approx -\alpha_{t}\nabla \left(\bar{\rho c_{p}T}\right)≃-k_{t}\nabla \bar{T}\\ \bar{j_{c,t}} & =\bar{u^{\prime }C_{c}^{\prime }}\; & \; & \approx -D_{c,t}\nabla \bar{C_{c}}\\ \bar{i_{k,t}} & =\bar{u^{\prime }{\left(\frac{z_{k}^{2}F^{2}C_{k}\Phi }{RT}\right)}^{\prime }}\; & \; & \approx -D_{k,t}\nabla \bar{\left(\frac{z_{k}^{2}F^{2}C_{k}\Phi }{RT}\right)}≃-\kappa_{k,t}\nabla \bar{\Phi } \end{array} \end{array}$$

where $\alpha_{t}$ is the thermal eddy dispersion coefficient (or eddy diffusivity) [m${}^{2}$ s${}^{-1}$], $k_{t}$ is the thermal eddy conductivity [J K${}^{-1}$ m${}^{-1}$ s${}^{-1}$], $D_{c,t}$ is the eddy dispersion coefficient for the *c*th chemical species [m${}^{2}$ s${}^{-1}$], $D_{k,t}$ is the eddy dispersion coefficient for the *k*th ion [m${}^{2}$ s${}^{-1}$] and $\kappa_{k,t}$ is the electrical eddy conductivity for the *k*th ion [A V${}^{-1}$ m${}^{-1}$] [65,77,145].

Now consider the effect of turbulence on the local entropy production for an isolated diffusion process (35)–(38), generalized as ${\hat{\dot{\sigma }}}_{D_{X}}=j_{X}\cdot \nabla Y$, where $j_{X}=uC_{X}$ is the flux of *X*, $C_{X}$ is the concentration of *X*, $D_{X}$ is the diffusion coefficient for *X* and ∇Y is the gradient in *Y* conjugate to *X*. Applying the Reynolds decomposition and averaging gives:

(89) σ˙^DX¯=−jX¯·∇Y¯−jX′¯·∇Y¯−jX¯·(∇Y)′¯−jX′·(∇Y)′¯=−jX¯·∇Y¯−u′CX′¯·∇Y¯−uC¯·CX′(∇Y)′¯−CX¯u′·(∇Y)′¯−CX′u′·(∇Y)′¯

using ab¯b¯¯=ab¯b¯ for means, $\bar{a^{\prime }}=0$ for isolated terms and $\bar{\nabla Y}=\nabla \bar{Y}$ for gradients [65,93]. As evident, the mean entropy production is complicated by the presence of diadic and triadic Reynolds terms [59]. The eddy coefficient correlations (87) and (88) only reduce the second term in the last line of (89), with the remaining Reynolds terms unresolved. If the fluxes or gradients are substituted by diffusion equations in the manner of (39)–(42), or if there are cross-phenomena (65), even more complicated Reynolds terms will be generated [146].

Many authors adopt simplified closure models for (89) based only on $\bar{{\hat{\dot{\sigma }}}_{D_{X,t}}}=-\bar{u^{\prime }C_{X}^{\prime }}\cdot \nabla \bar{Y}$, neglecting the other Reynolds terms. Applying entropic similarity by analogy with (43) and (44), and substituting (87), (88) and constant mean gradients, these give:

(90) $$\begin{array}{ccccc} {\hat{\Pi }}_{\nu_{t}/\alpha_{t}}\; & =\frac{\bar{{\hat{\dot{\sigma }}}_{\nu_{t}}}}{\bar{{\hat{\dot{\sigma }}}_{\alpha_{t}}}}\to Pr_{t}=\frac{\nu_{t}}{\alpha_{t}},\; & {\hat{\Pi }}_{\nu_{t}/D_{c,t}}\; & =\frac{\bar{{\hat{\dot{\sigma }}}_{\nu_{t}}}}{\bar{{\hat{\dot{\sigma }}}_{D_{c,t}}}}\to Sc_{c,t}=\frac{\nu_{t}}{D_{c,t}},\; & \\ {\hat{\Pi }}_{\nu_{t}/D_{k,t}}\; & =\frac{\bar{{\hat{\dot{\sigma }}}_{\nu_{t}}}}{\bar{{\hat{\dot{\sigma }}}_{D_{k,t}}}}\to Sc_{k,t}=\frac{\nu_{t}}{D_{k,t}},\; & {\hat{\Pi }}_{\alpha_{t}/D_{c,t}}\; & =\frac{\bar{{\hat{\dot{\sigma }}}_{\alpha_{t}}}}{\bar{{\hat{\dot{\sigma }}}_{D_{c,t}}}}\to Le_{c,t}=\frac{\alpha_{t}}{D_{c,t}},\; & \\ {\hat{\Pi }}_{\alpha_{t}/D_{k,t}}\; & =\frac{\bar{{\hat{\dot{\sigma }}}_{\alpha_{t}}}}{\bar{{\hat{\dot{\sigma }}}_{D_{k,t}}}}\to Le_{k,t}=\frac{\alpha_{t}}{D_{k,t}},\; & {\hat{\Pi }}_{D_{c,t}/D_{k,t}}\; & =\frac{\bar{{\hat{\dot{\sigma }}}_{D_{c,t}}}}{\bar{{\hat{\dot{\sigma }}}_{D_{k,t}}}}\to \frac{D_{c,t}}{D_{k,t}},\; & \\ {\hat{\Pi }}_{D_{c,t}/D_{b,t}}\; & =\frac{\bar{{\hat{\dot{\sigma }}}_{D_{c,t}}}}{\bar{{\hat{\dot{\sigma }}}_{D_{b,t}}}}\to \frac{D_{c,t}}{D_{b,t}},\; & {\hat{\Pi }}_{D_{k,t}/D_{\ell ,t}}\; & =\frac{\bar{{\hat{\dot{\sigma }}}_{D_{k,t}}}}{\bar{{\hat{\dot{\sigma }}}_{D_{\ell ,t}}}}≃\frac{z_{k}^{2}C_{k}D_{k,t}}{z_{\ell }^{2}C_{\ell }D_{\ell ,t}}\to \frac{D_{k,t}}{D_{\ell ,t}}\; \end{array}$$

These respectively give the turbulent Prandtl, Schmidt (species), Schmidt (charge), Lewis (species) and Lewis (charge) numbers, and ratios of eddy dispersion coefficients for different chemical or charged species [15,65,77,93]. By the “Reynolds analogy”, some authors argue these should be constant for fixed turbulent conditions [93]. The same groups can also be obtained from ratios of the turbulent entropy fluxes, analogous to those in (47) and (48).

An additional family of groups can be obtained from ratios of the turbulent and mean-product entropy production terms—the second and first terms in (89)—reducing to ratios of the turbulent dispersion and molecular diffusion coefficients:

(91) $$\begin{array}{c} \begin{array}{cc} {\hat{\Pi }}_{\nu_{t}/\nu }\; & =\frac{\bar{{\hat{\dot{\sigma }}}_{\nu_{t}}}}{\bar{{\hat{\dot{\sigma }}}_{\nu }}}\to \frac{\nu_{t}}{\nu },\;{\hat{\Pi }}_{\alpha_{t}/\alpha }=\frac{\bar{{\hat{\dot{\sigma }}}_{\alpha_{t}}}}{\bar{{\hat{\dot{\sigma }}}_{\alpha }}}\to \frac{\alpha_{t}}{\alpha },\;{\hat{\Pi }}_{D_{c,t}/D_{c}}=\frac{\bar{{\hat{\dot{\sigma }}}_{D_{c,t}}}}{\bar{{\hat{\dot{\sigma }}}_{D_{c}}}}\to \frac{D_{c,t}}{D_{c}},\;\\ {\hat{\Pi }}_{D_{k,t}/D_{k}}\; & =\frac{\bar{{\hat{\dot{\sigma }}}_{D_{k,t}}}}{\bar{{\hat{\dot{\sigma }}}_{D_{k}}}}\to \frac{D_{k,t}}{D_{k}} \end{array} \end{array}$$

The first group is similar to the Reynolds number (1) or (74), but defined locally, while the remaining three give local analogs of the Péclet numbers $Pe_{\alpha }$, $Pe_{c}$ and $Pe_{k}$, respectively (86). If there are thermodynamic cross-phenomena, analogous groups can be defined by entropic similarity using the Onsager relations (65) and (66):

(92) $$\begin{array}{c} \begin{array}{c} {\hat{\Pi }}_{L_{c,d,t}/L_{a,b,t}}=\frac{\bar{{\hat{\dot{\sigma }}}_{L_{c,d,t}}}}{\bar{{\hat{\dot{\sigma }}}_{L_{a,b,t}}}}\to \frac{L_{c,d,t}}{L_{a,b,t}},\;{\hat{\Pi }}_{L_{a,b,t}/L_{a,b}}=\frac{\bar{{\hat{\dot{\sigma }}}_{L_{a,b,t}}}}{\bar{{\hat{\dot{\sigma }}}_{L_{a,b}}}}\to \frac{L_{a,b,t}}{L_{a,b}} \end{array} \end{array}$$

where $L_{ı,j,t}$ is the turbulent phenomenological coefficient for the (ı,j)th process [m${}^{2}$ s${}^{-1}$].

### 5.3. Convective Dispersion

For heat or mass transfer processes involving fluid flow past a solid surface or fluid interface, it is necessary to consider the combined effect of diffusion and bulk fluid motion, referred to as *convection* [15,16,58,77,80,81,92,93,94,95,147,148,149,150]. Convection can be further classified into *forced convection*, due to fluid flow under a pressure gradient, and *free* or *natural convection*, due to fluid flow caused by heat-induced differences in temperature and density. Examples include heat exchange, extraction, sorption, drying and membrane filtration and latent heat exchange processes such as evaporation, distillation and condensation. Convection also arises in charge transfer such as electrolysis [151]. Convection processes are commonly analyzed by the linear transport relations:

(93) $$\begin{array}{cccccc} \left|\right|\tilde{j_{Q}}\left|\right| & =h_{Q}\Delta T,\; & \left|\right|\tilde{j_{c}}\left|\right| & =h_{c}\Delta \chi_{c}={\tilde{h}}_{c}\Delta C_{c},\; & \left|\right|\tilde{i_{k}}\left|\right| & =h_{k}\Delta \Phi \end{array}$$

where $\tilde{j_{X}}$ is the convective flux of *X* normal to the boundary, $h_{Q}$ is the heat transfer coefficient [J K${}^{-1}$ m${}^{-2}$ s${}^{-1}$], $h_{c}$ is the mass transfer coefficient for the *c*th species [mol m${}^{-2}$ s${}^{-1}$], ${\tilde{h}}_{c}$ is the mass transfer film coefficient for the *c*th species [m s${}^{-1}$], $h_{k}$ is the charge transfer coefficient for the *k*th ion [C V${}^{-1}$ m${}^{-2}$ s${}^{-1}$], $\chi_{c}=C_{c}/C$ is the mole fraction [–], $C=\sum_{c}C_{c}$ is the total concentration [mol m${}^{-3}$] and Δ represents a difference between two phases. The transfer coefficients are specific to each process and flow geometry.

We now apply the principle of entropic similarity to examine the transport regime during convection, based on ratios of entropy fluxes for convection and diffusion processes analogous to (49). Using (47) for fixed intensive variables, substituting the convective fluxes (93) and diffusive fluxes (24), (26) and (27) gives the entropic groups:

(94) $$\begin{array}{cccc} {\hat{\Pi }}_{h_{Q}/\alpha }\; & =\frac{\left|\right|j_{S,h_{Q}}\left|\right|}{\left|\right|j_{S,\alpha }\left|\right|}=\frac{\left|\right|\tilde{j_{Q}}\left|\right|\frac{1}{T}}{\left|\right|j_{Q}\left|\right|\frac{1}{T}}=\frac{h_{Q}\Delta T}{k||\nabla T||}\; & \; & \to Nu=\frac{h_{Q}d_{Q}}{k} \end{array}$$

(95) $$\begin{array}{cccc} {\hat{\Pi }}_{h_{c}/D_{c}}\; & =\frac{\left|\right|j_{S,h_{c}}\left|\right|}{\left|\right|j_{S,D_{c}}\left|\right|}=\frac{\left|\right|\tilde{j_{c}}\left|\right|\frac{\mu_{c}}{T}}{\left|\right|j_{c}\left|\right|\frac{\mu_{c}}{T}}=\frac{h_{c}\Delta \chi_{c}}{D_{c}||\nabla C_{c}\left|\right|}=\frac{{\tilde{h}}_{c}\Delta C_{c}}{D_{c}||\nabla C_{c}\left|\right|}\; & \; & \to Sh_{c}=\frac{h_{c}d_{c}}{CD_{c}}=\frac{{\tilde{h}}_{c}d_{c}}{D_{c}} \end{array}$$

(96) $$\begin{array}{cccc} {\hat{\Pi }}_{h_{k}/D_{k}}\; & =\frac{\left|\right|j_{S,h_{k}}\left|\right|}{\left|\right|j_{S,D_{k}}\left|\right|}=\frac{\left|\right|\tilde{i_{k}}\left|\right|\frac{\Phi }{T}}{\left|\right|i_{k}\left|\right|\frac{\Phi }{T}}=\frac{h_{k}\Delta \Phi }{D_{k}\left||\nabla \Phi |\right|}\; & \; & \to Sh_{k}=\frac{h_{k}d_{k}}{\kappa_{k}}≃\frac{RTh_{k}d_{k}}{z_{k}^{2}F^{2}C_{k}D_{k}} \end{array}$$

where $d_{Q},d_{c}$ and $d_{k}$ are length scales [m] arising from each normalized gradient, Nu is the Nusselt number and $Sh_{c}$ and $Sh_{k}$ are the chemical and charge Sherwood numbers. Traditionally, Nu and $Sh_{c}$ are derived by kinematic similarity from the ratios of convective and molecular fluxes of heat or chemical species (see above references). Nu can also be shown to represent the dimensionless temperature gradient from the surface or interface [15,16,93,94,95], while $Sh_{c}$ is the dimensionless concentration gradient [15,95]. By the same reasoning, $Sh_{k}$ can be interpreted as the dimensionless electrical potential gradient from the surface or interface.

In forced convection, Nu is commonly expressed as a function of Re and Pr for a given flow geometry, while $Sh_{c}$ is correlated with Re and $Sc_{c}$ (see above references). In free convection due to heat transfer, the inertia arises from temperature-induced differences in density, giving the velocity scale $U_{\mathrm{conv}}=\sqrt{gd|\Delta \rho |/\rho }$ [m s${}^{-1}$], where *d* is a length scale [m] and |Δρ| is the magnitude of the density difference between the wall and free-stream fluid [kg m${}^{-3}$] [16]. For an internal flow, comparing (72) and (86), we define the entropic groups:

(97) $$\begin{array}{c} \begin{array}{cc} \Pi_{\mathrm{int},I/\nu }^{\prime }\; & ={\left(\frac{{\dot{\sigma }}_{\mathrm{int},I}}{{\dot{\sigma }}_{\mathrm{int},\nu }}\right)}^{2}∼{\left(\frac{f\;U_{\mathrm{conv}}d}{\nu }\right)}^{2}=f^{2}Gr,\\ \mathrm{with}\;\;\; & Gr={\left(\frac{U_{\mathrm{conv}}d}{\nu }\right)}^{2}=\frac{gd^{3}\left|\Delta \rho \right|}{\rho \nu^{2}}=\frac{g\beta d^{3}\left|\Delta T\right|}{\nu^{2}} \end{array} \end{array}$$

(98) $$\begin{array}{c} \begin{array}{cc} \Pi_{\mathrm{int},I/\alpha ,\nu }^{\prime }\; & =\frac{{\dot{\sigma }}_{\mathrm{int},I}^{2}}{{\dot{\sigma }}_{\mathrm{int},\nu }\;{\dot{\sigma }}_{\mathrm{int},\alpha }}∼f^{2}Ra,\;\\ \mathrm{with}\;\;\; & Ra=\frac{{\left(U_{\mathrm{conv}}d\right)}^{2}}{\nu \alpha }=\frac{gd^{3}\left|\Delta \rho \right|}{\rho \nu \alpha }=\frac{g\beta d^{3}\left|\Delta T\right|}{\nu \alpha }=GrPr \end{array} \end{array}$$

using Δρ≃−ρβΔT, where Gr is the Grashof number, Ra is the Rayleigh number and β is the thermal expansion coefficient [K${}^{-1}$] (see above references). For boundary layer flows, analogs of (97) and (98) containing $C_{D}$ rather than *f* are required, in which Gr and Ra are functions of position. As evident, Gr is the square ratio of the inertial dispersion to viscous diffusion coefficients in a buoyancy-driven flow, while Ra is a composite group based on the inertial dispersion, viscous and heat diffusion coefficients. Traditionally, Gr is interpreted by dynamic similarity as the ratio of buoyancy to viscous forces, while Ra is a composite ratio of buoyancy, viscous and heat transport forces. In free convection, Nu is generally expressed as a function of Gr (or Ra), Pr and geometry, while $Sh_{c}$ is correlated as a function of Gr, $Sc_{c}$ and geometry (see above references). Bejan [149] uses a length-scale analysis to argue for correlations based on Ra rather than Gr, with a Boussinesq number Bq=RaPr for low-Pr fluids. The Richardson number $Ri=Gr/Re^{2}$ can be used to characterize the flow regime as free (Ri≫1), forced (Ri≪1) or mixed (Ri∼1) convection [16,93,94,95,150].

In free convection due to chemical gradients, mass-transfer analogs of Gr and Ra are defined by the |Δρ| forms in (97) and (98) [95]. For variations in salinity S [-], applying $\Delta \rho ≃\rho \beta^{\prime }\Delta S$ gives a salinity Rayleigh number $Rs=gd^{3}\beta^{\prime }\left|\Delta S\right|/\nu \alpha$, where $\beta^{\prime }$ is a haline contraction coefficient [-] [152,153].

For many processes, more comprehensive formulations of the groups in (94)–(98) may be necessary. For example, in heat transfer systems, there may be multiple driving temperatures in ${\hat{\Pi }}_{h_{Q}/\alpha }$ [150], while in chemical systems, it may be necessary to introduce chemical activities into ${\hat{\Pi }}_{h_{c}/D_{c}}$ or $Sh_{c}$ [77]. Unsteady convection processes require an extended analysis with different length and time scales [77]. Double diffusion—such as of heat and salinity—can induce entropy-producing instabilities, analyzed by both Ra and Rs or a composite group [152,153,154]. For convection with chemical reaction, it is necessary to consider the three-way competition between diffusion, convection and chemical reaction processes [80]; this may require the synthesis of groups from (60) or (62) with those in (94)–(96). With thermodynamic cross-phenomena (Section 4.3), it is necessary to include the total diffusive fluxes (65) in (94)–(96) rather than those based on individual mechanisms.

The above analyses lead to a plethora of entropic groups for heat and mass transfer, examined in Appendix C. Analogous groups can be defined for the convection of charge.

### 5.4. Hydrodynamic Dispersion

For flow in porous media such as groundwater flow or in a packed bed, the flow regime is usually not turbulent except under high hydraulic gradients. However, other mixing mechanisms arise in the presence of the porous medium. These include:

(i) *Molecular diffusion*, due to the random motions of molecules. This is represented by Fick’s law (26) but corrected to account for blocking by solid particles [155,156]:
  (99) $$\begin{array}{cc} j_{c} & =-D_{c}^{*}\nabla C_{c},\;\mathrm{with}\;\;D_{c}^{*}=\omega D_{c} \end{array}$$
  where $D_{c}^{*}$ is the bulk molecular diffusion coefficient for the *c*th species [m${}^{2}$ s${}^{-1}$] and ω<1 is a correction factor [-]. Some authors correlate ω≈ϵφ/ϑ, where ϵ is the porosity, φ is a pore constriction factor and ϑ is the tortuosity [80].
(ii) *Mechanical dispersion*, due to the physical motion of fluid around solid obstacles, causing spreading in the longitudinal and transverse directions [155,156,157]. This is generally represented by a Fick’s law relation in each *i*th direction [14,155,156]:
  (100) $$\begin{array}{c} \begin{array}{cc} j_{c,ı} & =-D_{c,ı}^{m}\frac{\partial C_{c}}{\partial ı},\;\mathrm{with}\;\;D_{c,ı}^{m}=Ua_{ı} \end{array} \end{array}$$
  where $D_{c,ı}^{m}$ is the mechanical dispersion coefficient for the *c*th species [m${}^{2}$ s${}^{-1}$]. Commonly, this is further correlated with the superficial fluid velocity *U* [m s${}^{-1}$], to define the dispersivity $a_{ı}$ [m].

Collectively, the two spreading processes are termed *hydrodynamic dispersion*, represented by an overall hydrodynamic dispersion coefficient $D_{c,ı}$ [m${}^{2}$ s${}^{-1}$] [14,155,156,158]:

(101) $$\begin{array}{c} \begin{array}{c} D_{c,ı}=D_{c,ı}^{m}+D_{c}^{*}=Ua_{ı}+D_{c}^{*} \end{array} \end{array}$$

An analogous relation can be written for the *k*th charged species. For heat, it is necessary to account for conduction through the solid *s* and fluid *f* [14,155]:

(102) $$\begin{array}{c} \begin{array}{cc} a_{ı} & =a_{ı}^{m}+\alpha^{*}=Ua_{ı}+\alpha^{*},\;\mathrm{with}\;\;\alpha^{*}=\epsilon \alpha_{f}+\left(1-\epsilon \right)\alpha_{s} \end{array} \end{array}$$

where $\alpha^{*}$, $a_{ı}^{m}$ and $a_{ı}$ are the bulk diffusion, mechanical and hydrodynamic dispersion coefficients for heat [m${}^{2}$ s${}^{-1}$].

Applying entropic similarity, the relative importance of mechanical dispersion and diffusion can be represented by the hydrodynamic Péclet numbers [14,159]:

(103) $$\begin{array}{c} \begin{array}{cc} {\hat{\Pi }}_{D_{c,ı}}\; & =\frac{{\hat{\dot{\sigma }}}_{D_{c,ı}^{m}}}{{\hat{\dot{\sigma }}}_{D_{c}^{*}}}\to Pe_{D_{c,ı}}=\frac{D_{c,ı}^{m}}{D_{c}^{*}}=\frac{Ua_{ı}}{D_{c}^{*}},\;{\hat{\Pi }}_{a_{ı}}=\frac{{\hat{\dot{\sigma }}}_{a_{ı}^{m}}}{{\hat{\dot{\sigma }}}_{\alpha^{*}}}\to Pe_{a_{ı}}=\frac{a_{ı}^{m}}{\alpha^{*}}=\frac{Ua_{ı}}{\alpha^{*}},\\ {\hat{\Pi }}_{D_{k,ı}}\; & =\frac{{\hat{\dot{\sigma }}}_{D_{k,ı}^{m}}}{{\hat{\dot{\sigma }}}_{D_{k}^{*}}}\to Pe_{D_{k,ı}}=\frac{D_{k,ı}^{m}}{D_{k}^{*}}=\frac{Ua_{ı}}{D_{k}^{*}} \end{array} \end{array}$$

These can also be defined using total hydrodynamic dispersion coefficients such as $D_{c,ı}/D_{c}^{*}$, or as ratios of inertial and diffusion terms $U\ell /D_{c}^{*}$, where *ℓ* is a length scale [m] [14,155,156]. For contaminant migration in clay soils, generally $Pe_{D_{c,ı}}\ll 1$, dominated by diffusion, while for sands and gravels $Pe_{D_{c,ı}}\gg 1$, dominated by mechanical dispersion. Additional groups can be defined for competition with chemical reactions, analogous to (59) and (60), or entropy fluxes, analogous to (49).

### 5.5. Shear-Flow Dispersion

An additional mixing mechanism in internal and open channel flows is *shear-flow dispersion*, arising from the difference in fluid velocities between the centerline and solid walls [15,158,160,161]. Employing a Reynolds decomposition $a=\langle a\rangle +a^{\prime \prime }$ based on the cross-sectional average 〈a〉 and deviation $a^{\prime \prime }$ rather than a temporal decomposition, this is correlated as:

(104) $$\begin{array}{c} \langle j_{c,x,\mathrm{shear}}\rangle =\langle u^{\prime \prime }C_{c}^{\prime \prime }\rangle \approx -K\frac{\partial \langle C_{c}\rangle }{\partial x} \end{array}$$

where, for the *c*th species and downstream direction *x*, $\langle j_{c,x,\mathrm{shear}}\rangle$ is the mean flux relative to the flow [mol m${}^{-2}$ s${}^{-1}$], $\langle u^{\prime \prime }C_{c}^{\prime \prime }\rangle$ is the mean Reynolds flux [mol m${}^{-2}$ s${}^{-1}$], *K* is the shear dispersion coefficient [m${}^{2}$ s${}^{-1}$] and $\langle C_{c}\rangle$ is the mean concentration [mol m${}^{-3}$]. Applying entropic similarity using (104) and cross-sectional averages of (48) or (88) yields:

(105) $$\begin{array}{cc} {\hat{\Pi }}_{K/D_{c}}=\frac{\langle {\hat{\dot{\sigma }}}_{K}\rangle }{\langle {\hat{\dot{\sigma }}}_{D_{c}}\rangle }\to \frac{K}{D_{c}},\;{\hat{\Pi }}_{K/D_{c,t}}\; & =\frac{\langle {\hat{\dot{\sigma }}}_{K}\rangle }{\langle {\hat{\dot{\sigma }}}_{D_{c,t}}\rangle }\to \frac{K}{D_{c,t}} \end{array}$$

In many natural water bodies such as rivers and estuaries $K\gg D_{c,t}\gg D_{c}$, for which ${\hat{\Pi }}_{K/D_{c}}\gg {\hat{\Pi }}_{K/D_{c,t}}\gg 1$ [15,160].

### 5.6. Dispersion of Bubbles, Drops and Particles

Consider a system containing a dispersed phase composed of bubbles, drops or solid particles of density $\rho_{d}$ [kg m${}^{-3}$] and length scale *d* [m] within a continuous fluid of density $\rho_{c}$ [kg m${}^{-3}$] and kinematic viscosity $\nu_{c}$ [m${}^{2}$ s${}^{-1}$]. From the analysis of convection in Section 5.3, the difference in densities creates a buoyancy-driven inertia between the phases, represented by the velocity scale $U_{\mathrm{disp}}=\sqrt{gd|\Delta \rho |/\rho_{c}}$ [m s${}^{-1}$], where $\left|\Delta \rho |=\right|\rho_{d}-\rho_{c}|$. For external flow around dispersed phase particles with drag coefficient $C_{D}$, the ratio of the entropy production by intrinsic to external inertial dispersion (77), or to viscous diffusion (78), gives:

(106) $$\begin{array}{c} \begin{array}{cccc} \Pi_{\mathrm{ext},I/\mathrm{disp},I}\; & =\frac{{\dot{\sigma }}_{\mathrm{ext},I}}{{\dot{\sigma }}_{\mathrm{disp},I}}∼Fr_{\mathrm{disp}}^{3}\; & \mathrm{with}\;Fr_{\mathrm{disp}}\; & =\frac{U}{U_{\mathrm{disp}}}=\frac{U}{\sqrt{gd|\Delta \rho |/\rho_{c}}}\\ \Pi_{\mathrm{disp},I/\mathrm{ext},\nu_{c}}\; & ={\left(\frac{{\dot{\sigma }}_{\mathrm{disp},I}}{{\dot{\sigma }}_{\mathrm{ext},\nu_{c}}}\right)}^{2}∼C_{D}^{2}ArFr_{\mathrm{disp}}^{-4}\; & \mathrm{with}\;\;Ar\; & ={\left(\frac{U_{\mathrm{disp}}d}{\nu_{c}}\right)}^{2}=\frac{gd^{3}\left|\Delta \rho \right|}{\rho_{c}\nu_{c}^{2}} \end{array} \end{array}$$

where $Fr_{\mathrm{disp}}$ is the densimetric particle Froude number and Ar is the Archimedes number [119,128,162,163]. As evident, $Fr_{\mathrm{disp}}$ is the ratio of inertial dispersion by external to intrinsic sources, traditionally interpreted by dynamic similarity as the ratio of inertial to buoyancy forces. Ar is of the same form as Gr (97), traditionally interpreted as buoyancy relative to viscous forces. Comparing (98), we identify $Fr_{\mathrm{disp}}^{2}=Re^{2}/Ar=RePe_{\alpha }/Ra=Ri^{-1}$, for Ra and Ri now defined using $U_{\mathrm{disp}}$. The above groups—often rewritten in terms of the friction velocity $u^{*}$ (Section 5.1) instead of *U*—are widely used for the analysis of dispersed phase entrainment, transport and sediment bed forms [164,165,166,167].

For drops or bubbles with surface or interfacial tension ς [J m${}^{-2}$], the external or intrinsic rates of entropy production needed to maintain the dispersed phase are:

(107) $$\begin{array}{c} {\dot{\sigma }}_{\mathrm{ext},\varsigma }=\frac{A_{d}\varsigma }{T\theta_{\mathrm{ext}}}∼\frac{\varsigma Ud}{T},\;{\dot{\sigma }}_{\mathrm{disp},\varsigma }=\frac{A_{d}\varsigma }{T\theta_{\mathrm{disp}}}∼\frac{\varsigma U_{\mathrm{disp}}d}{T} \end{array}$$

defined for two choices of time scale $\theta_{\mathrm{ext}}∼d/U$ or $\theta_{\mathrm{disp}}∼d/U_{\mathrm{disp}}$ [s], where $A_{d}∼d^{2}$ is the surface area [m${}^{2}$]. Applying entropic similarity to combinations of the intrinsic or external inertial dispersion (77), viscous diffusion (78) and tension (107) gives the groups:

(108) $$\begin{array}{cc} \Pi_{\mathrm{ext},I/\mathrm{ext},\varsigma }\; & =\left(\frac{{\dot{\sigma }}_{\mathrm{ext},I}}{{\dot{\sigma }}_{\mathrm{ext},\varsigma }}\right)∼C_{D}We\;\mathrm{with}\;\;We=\frac{\rho_{c}U^{2}d}{\varsigma }=EoFr_{\mathrm{disp}}^{2} \end{array}$$

(109) $$\begin{array}{cc} \Pi_{\mathrm{disp},I/\mathrm{disp},\varsigma }\; & =\left(\frac{{\dot{\sigma }}_{\mathrm{disp},I}}{{\dot{\sigma }}_{\mathrm{disp},\varsigma }}\right)∼C_{D}Eo\;\mathrm{with}\;\;Eo=\frac{\rho_{c}U_{\mathrm{disp}}^{2}d}{\varsigma }=\frac{gd^{2}\left|\Delta \rho \right|}{\varsigma } \end{array}$$

(110) $$\begin{array}{cc} \Pi_{\mathrm{ext},\nu_{c}/\mathrm{ext},\varsigma }\; & =\left(\frac{{\dot{\sigma }}_{\mathrm{ext},\nu_{c}}}{{\dot{\sigma }}_{\mathrm{ext},\varsigma }}\right)∼Ca=\frac{\rho_{c}\nu_{c}U}{\varsigma } \end{array}$$

where We is the Weber number, Eo is the Eötvös or Bond number and Ca is the capillary number. These and the Morton number $Mo=g\nu_{c}^{4}\rho_{c}^{2}\left|\Delta \rho \right|/\varsigma^{3}=Eo^{3}/Ar^{2}$ are widely used to characterize bubble and droplet shapes, flow regimes and their entrapment in porous media [128,156].

## 6. Universal Diffusion Processes

As a final topic, consider the common representation of the universe based on five dimensions, represented by the set of fundamental SI units {kg, m, s, K, A}. By dimensional considerations, these must be represented by five universal constants, commonly taken as:

1. c=299792458 m s${}^{-1}$, the speed of light in a vacuum;
2. $\hbar =1.054\;571\;817\times 10^{-34}$ J s (or kg m${}^{2}$ s${}^{-1}$), the reduced Planck constant;
3. $G=6.67430\times 10^{-11}$ m${}^{3}$ kg${}^{-1}$ s${}^{-2}$, the gravitational constant;
4. $e=1.602\;176\;634\times 10^{-19}$ C (or A s), the elementary positive charge, and
5. $k_{B}=1.380\;649\times 10^{-23}$ J K${}^{-1}$ (or kg m${}^{2}$ s${}^{-2}$ K${}^{-1}$), the Boltzmann constant,

written in the 2019 redefinition of SI units [168]. These can be used to define natural or Planck units of mass, length, time, temperature, force and other quantities by dimensional reasoning [169]. They also define a universal diffusion coefficient:

(111) $$\begin{array}{c} D_{\mathrm{univ}}∼\sqrt{\frac{\hbar G}{c}}=4.845\;410\;655\times 10^{-27}\;m{}^{2}\;s{}^{-1} \end{array}$$

Equation (111) expresses a minimum bound for diffusion processes in the universe. It also suggests an additional conjugate pair for Heisenberg’s uncertainty principle $\delta m\;\delta D\geq \frac{1}{2}\hbar$ based on the uncertainties in the mass δm and diffusion coefficient δD of a physical particle.

Applying entropic similarity, we can construct universal dimensionless groups to compare molecular diffusion processes with universal diffusion. For the diffusion of heat, momentum, species *c* or charged particle *k* under constant gradients, this gives:

(112) $$\begin{array}{c} \begin{array}{cccc} {\hat{\Pi }}_{\alpha /D_{\mathrm{univ}}}\; & =\frac{{\hat{\dot{\sigma }}}_{\alpha }}{{\hat{\dot{\sigma }}}_{D_{\mathrm{univ}}}}∼\frac{\alpha }{D_{\mathrm{univ}}},\; & {\hat{\Pi }}_{\nu /D_{\mathrm{univ}}}\; & =\frac{{\hat{\dot{\sigma }}}_{\nu }}{{\hat{\dot{\sigma }}}_{D_{\mathrm{univ}}}}∼\frac{\nu }{D_{\mathrm{univ}}},\\ {\hat{\Pi }}_{D_{c}/D_{\mathrm{univ}}}\; & =\frac{{\hat{\dot{\sigma }}}_{D_{c}}}{{\hat{\dot{\sigma }}}_{D_{\mathrm{univ}}}}∼\frac{D_{c}}{D_{\mathrm{univ}}},\; & {\hat{\Pi }}_{D_{k}/D_{\mathrm{univ}}}\; & =\frac{{\hat{\dot{\sigma }}}_{D_{k}}}{{\hat{\dot{\sigma }}}_{D_{\mathrm{univ}}}}∼\frac{D_{k}}{D_{\mathrm{univ}}} \end{array} \end{array}$$

In a typical natural or engineered system, these will be strongly dominated by molecular diffusion, but in small or quantum systems the universal dispersion may be important.

## 7. Conclusions

This study proposes an additional category of dimensionless groups based on the principle of *entropic similarity*, involving ratios of entropic terms. Since all processes involving work against friction, dissipation, diffusion, dispersion, mixing, separation, chemical reaction, gain of information or other irreversible changes are driven by (or must overcome) the second law of thermodynamics, it is appropriate to analyze these processes directly in terms of competing entropy-producing and transporting phenomena and the dominant entropic regime, rather than indirectly in terms of their associated forces. The theoretical foundations of entropy are examined in Section 2, following which the principle of entropic similarity is established in Section 3, to give three definitions of an entropic dimensionless group: (i) a ratio of entropy production terms; (ii) a ratio of entropy flow rates or fluxes; or (iii) an information-theoretic definition based on a ratio of information fluxes. These definitions are used to derive entropic groups for a number of entropy-producing and transporting phenomena relevant to fluid mechanics, chemical and environmental engineering, including diffusion and chemical reaction processes (Section 4), a variety of dispersion mechanisms (Section 5) and diffusion in the universe (Section 6). Comparing the derived entropic dimensionless groups to those obtained by kinematic or dynamic similarity, or by other means, we can draw several conclusions:

1. For many groups defined by dynamic similarity as ratios of forces $\Pi =F_{1}/F_{2}$ (19), their reformulation in terms of macroscopic entropy production terms $\Pi ={\dot{\sigma }}_{1}/{\dot{\sigma }}_{2}$ (20) recovers the same or a similar dimensionless group. Examples in this category include the Reynolds numbers for internal, external or rotational flows (Section 5.1), the Péclet numbers for diffusion (Section 5.1), the Grashof and Rayleigh numbers in convection (Section 5.3) and the Weber and Eötvös or Bond numbers for bubbles and droplets (Section 5.6). However, in all these cases, the entropic formulation recovers the product of a friction factor, drag or torque coefficient (or its square) and the dimensionless group, rather than the standalone group given by dynamic similarity. The Archimedes number (Section 5.3) is also obtained as a composite group. Furthermore, the entropic perspective provides an alternative interpretation of these groups: for example, the Reynolds number (1) can be reinterpreted as the ratio of the inertial dispersion and viscous diffusion coefficients, while the Grashof and Archimedes numbers (97) and (106) are the square of this ratio. Furthermore, the various Péclet numbers (86) express the ratio of the inertial dispersion coefficient to the heat, mass or charge diffusion coefficients, while the Rayleigh number (98) is a mixed ratio of inertial dispersion, viscous and heat diffusion coefficients. The Weber and Eötvös numbers are also directly obtained from the ratios of entropy production terms for inertial dispersion and surface or interfacial tension. The entropic dimensionless groups can also be used to construct entropic similarity diagrams such as those in Figure 2a,b and Figure 3a,b, providing a clear separation between the different entropic flow regimes present.
2. In contrast, many entropic groups defined by ratios of local entropy production terms $\hat{\Pi }={\hat{\dot{\sigma }}}_{1}/{\hat{\dot{\sigma }}}_{2}$ (20) or entropy fluxes $\hat{\Pi }=\left|\right|j_{S,1}\left|\right|/\left|\right|j_{S,2}\left|\right|$ (21) do not appear to have simple interpretations by dynamic similarity. These include ratios of diffusion terms leading to the Prandtl, Schmidt and Lewis numbers (Section 4.1), most chemical reaction groups including the Damköhler number (Section 4.2), cross-phenomenological groups (Section 4.3), ratios of turbulent dispersion coefficients (Section 5.2), the Péclet numbers for hydrodynamic dispersion (Section 5.4) and shear-flow dispersion groups (Section 5.5). Historically, such groups are commonly obtained directly by dimensional analysis or by non-dimensionalization of the governing equations rather than by similarity. The principle of entropic similarity therefore provides a more natural basis for their interpretation based on their entropic driving force. Furthermore, the entropic perspective yields extended formulations of these groups as well as many entirely new groups. Examples of the latter include the diffusion groups (43) and (49), hybrid diffusion groups containing a flux and a gradient such as (46), the group for diffusion relative to fluid entropy transport (49) and groups for chemical reaction relative to diffusion (60) or fluid transport (62).
3. A number of dimensionless groups admit multiple interpretations by entropic and other forms of similarity, leading to some interesting new insights. For example, the Nusselt and Sherwood numbers for convection processes (94)–(96), derived here by entropic similarity based on entropy fluxes, are traditionally obtained by kinematic similarity as ratios of convective and molecular fluxes of heat or chemical species. They can also be interpreted by geometric similarity as dimensionless temperature, concentration or electrical potential gradients [15,16,93,94,95]. Similarly, the densimetric particle Froude number (106) is derived here as the ratio of entropy production terms for inertial dispersion by external flow and the dispersed phase, but is traditionally interpreted by dynamic similarity as the ratio of inertial to buoyancy forces. It can also be identified as an inverse Richardson number, which distinguishes free and forced convection (Section 5.3). An equivalence between the Grashof and Archimedes numbers, as square ratios of inertial dispersion to viscous diffusion terms respectively for convection processes or a dispersed phase, is also established. Several other groups commonly used for heat or mass transfer (Appendix C), including the Biot, Fourier, Stefan, Eckert, Brinkman and Stanton numbers, can also be variously interpreted by kinematic, dynamic and entropic similarity.

To conclude, it is shown that the principle of entropic similarity enables the derivation of new dimensionless groups beyond those accessible by geometric, kinematic and dynamic similarity as well as the reinterpretation of many known dimensionless groups. These significantly expand the scope of dimensional analysis and similarity arguments for the resolution of new and existing problems across all branches of science and engineering.

Throughout this study, a concerted effort has been made to examine the transfer of electrical charge, to place this on an equal footing with the better-known relations for mass, momentum and energy transfer processes and chemical reactions. Charge transfer phenomena have important applications in electrolytic, electrochemical and photovoltaic processes—especially in the presence of fluid turbulence and convection—needed for the world energy transition from fossil fuels. Emphasis is also placed on the vector or tensor basis of the underlying physical phenomena, including velocities, forces, fluxes and gradients, which demands the use of a modern vector-tensor mathematical framework for the construction of dimensionless groups.

Finally, while this work examines a number of important entropic phenomena in mass, momentum, energy and charge transfer processes, chemical reactions and dispersion processes relevant to fluid flow systems, it is not claimed to be complete. Part II of this work [170] presents a separate analysis of the information-theoretic definition of similarity (22) and (23) and its application to wave phenomena. Many other important processes have not been examined from an entropic similarity perspective, including mixing and separation unit operations in chemical and environmental engineering [171], radioactive decay and nuclear processes [172], gravitation [173], hydraulic and hydrological systems [49,50], biological growth, evolutionary and planetary processes [29,48,174], transport systems [175,176] and economic systems and industrial ecology [177]. Further research is required on the derivation of entropic dimensionless groups to represent these and many other natural, engineered and human phenomena. 

## Data Availability

The data used in Figure 2 and Figure 3 were obtained from the sources cited in each figure caption.

## Appendix A. Electrochemical and Charge Carrier Relations

To formulate the charge density form of Ohm’s law (30), it is necessary to develop a theoretical formulation that accounts for both positive and negative ions—this is not evident in any individual reference found by the author. For the molar formulation, we first identify the charge flux for the *k*th species as $i_{k}=z_{k}Fj_{k}=z_{k}Fv_{k}C_{k}$, where $j_{k}$ is the molar flux [(mol species) m${}^{-2}$ s${}^{-1}$] and $v_{k}$ is the velocity [m s${}^{-1}$] of the *k*th species [78,79]. For negative ions $z_{k}<0$, we have $\mathrm{sign}(j_{k})<0$ and $\mathrm{sign}(v_{k})<0$ in a positive field, for which $\mathrm{sign}(i_{k})>0$; the sign reversal indicates that a flux of negative charge in the negative direction is equivalent to a flux of positive charge in the positive direction. The velocity can further be represented by $v_{k}=-z_{k}F\mu_{k}\nabla \Phi =-u_{k}\nabla \Phi$, where $\mu_{k}$ is the charged species mobility [(mol species) m${}^{2}$ J${}^{-1}$ s${}^{-1}$] and $u_{k}$ is the electric mobility [m${}^{2}$ V${}^{-1}$ s${}^{-1}$], connected by $u_{k}=z_{k}F\mu_{k}$ [78,79]. (Note the unfortunate overlap between the symbols commonly used for mobilities with other quantities defined in this study.) This yields $i_{k}=-z_{k}^{2}F^{2}\mu_{k}C_{k}\nabla \Phi =-z_{k}Fu_{k}C_{k}\nabla \Phi$ [79]. For negative ions $z_{k}<0$ we have $\mu_{k}>0$ and $u_{k}<0$, in a positive electric field sign(E)=−sign(∇Φ)>0, again giving $\mathrm{sign}(v_{k})<0$ and $\mathrm{sign}(i_{k})>0$. Finally, adopting the Sutherland–Einstein relation for ion diffusion $\mu_{k}=D_{k}/RT$ [178,179] gives $i_{k}=-z_{k}^{2}F^{2}D_{k}C_{k}\nabla \Phi /RT$ [85]. Moving all terms except $D_{k}$ inside the gradient—thus allowing for spatial inhomogeneity in the concentration and temperature—gives the first charge density relation in (30).

For the individual carrier formulation, we start with $i_{k}=n_{k}q_{k}v_{k}$ [84], hence $z_{k}Fc_{k}=n_{k}q_{k}$ and $i_{k}=-q_{k}^{2}n_{k}^{2}\mu_{k}\nabla \Phi /C_{k}$. For negative ions $q_{k}<0$, we again obtain $\mathrm{sign}(v_{k})<0$ and $\mathrm{sign}(i_{k})>0$ in a positive field. Substituting for the mobility $\mu_{k}$ and recognizing $C_{k}R=n_{k}k_{B}$ then gives $i_{k}=-q_{k}^{2}n_{k}D_{k}\nabla \Phi /k_{B}T$. Moving all terms except $D_{k}$ inside the gradient gives the second relation in (30).

## Appendix B. Thermodynamic Diffusion Relations

To reduce the thermodynamic diffusion parameter $D_{c}^{\prime }$ in (33), consider the relationship between the chemical potential $\mu_{c}$ [J (mol species)${}^{-1}$], (relative) chemical activity $\alpha_{c}$ [–] and specific molar concentration $m_{c}$ [(mol species) kg${}^{-1}$] for the *c*th species in solution [28,78,99,180]:

(A1) αc=expμc−μc◯RT=γcmcmc

where $\gamma_{c}$ is the activity coefficient [–] and ◯ refers to a quantity at a defined reference state. Commonly mc◯ 1 [(mol species) kg${}^{-1}$] is chosen to enable the cancellation of units [99]. Alternatively, for gaseous species:

(A2) αc=expμc−μc◯RT=fcpc◯=γ^cpc◯pc

where $f_{c}$ is the fugacity [Pa], ${\hat{\gamma }}_{c}$ is the fugacity coefficient [–] and $p_{c}=RTC_{c}$ is the partial pressure [Pa]. Rearranging and differentiating (A1) and (A2) for a spatially invariant reference state gives:

(A3) ∇mc=mc∇(μc−μc◯)RT−∇lnγc

(A4) ∇pc=pc∇(μc−μc◯)RT−∇lnγ^c

Substitution into (26) gives, respectively:

(A5) jc=−ρmcDc∇(μc−μc◯)RT−∇lnγc+∇lnρ

(A6) jc=−pcDcRT∇(μc−μc◯)RT−∇lnγ^c+∇ln1T

We see that the practical relation (26) conforms to the thermodynamic (33) for a spatially invariant (or small logarithmic gradients in the) activity or fugacity coefficient, fluid density and temperature, giving $D_{c}^{\prime }≃\rho m_{c}D_{c}/R=p_{c}D_{c}/R^{2}T$ respectively for solutes or gaseous species. Neglecting the spatial variation of activity or fugacity coefficients and temperature, the entropy production (41) becomes:

(A7) $$\begin{array}{c} \begin{array}{cc} {\hat{\dot{\sigma }}}_{D_{c}}\; & ≃\frac{\rho RD_{c}}{m_{c}}\;{\left|\left|\nabla m_{c}\right|\right|}^{2}=\frac{D_{c}}{p_{c}T}\;{\left|\left|\nabla p_{c}\right|\right|}^{2} \end{array} \end{array}$$

for a gradient-controlled system, respectively for diffusion in a liquid or a gas.

## Appendix C. Additional Entropic Groups in Heat and Mass Transfer

The analysis of convection in heat and mass transfer in Section 5.3 can be used to derive a number of related entropic dimensionless groups. For example, for “lumped system analysis” of the heating of a solid with surface area $A_{s}$ [m${}^{2}$], volume $V_{s}$ [m${}^{3}$] and length scale $\ell_{s}∼V_{s}/A_{s}$ [m], we obtain [81,150]:

(A8) $$\begin{array}{c} \begin{array}{cccc} \Pi_{h_{Q}/k_{s}}\; & =\frac{\left|\right|j_{S,h_{Q}}\left|\right|}{\left|\right|j_{S,\alpha_{s}}\left|\right|}=\frac{\left|\right|\tilde{j_{Q}}\left|\right|\frac{1}{T}}{\left|\right|j_{Q,s}\left|\right|\frac{1}{T}}=\frac{h_{Q}\Delta T}{k_{s}{||\left(\nabla T\right)}_{s}\left|\right|}\; & \; & \to Bi=\frac{h_{Q}\ell_{s}}{k_{s}},\\ \Pi_{k_{s}/{\left(\rho c_{p}\right)}_{s}}\; & =\frac{F_{S,\mathrm{cond}}^{\mathrm{net}}}{F_{S,\mathrm{stor}}^{\mathrm{net}}}=\frac{F_{Q,\mathrm{cond}}^{\mathrm{net}}\frac{1}{T}}{F_{Q,\mathrm{stor}}^{\mathrm{net}}\frac{1}{T}}=\frac{k_{s}{||\left(\nabla T\right)}_{s}\left|\right|A_{s}}{{\left(\rho c_{p}\right)}_{s}{\left(\Delta T\right)}_{s}V_{s}/t}\; & \; & \to Fo=\frac{k_{s}t}{\rho_{s}c_{p,s}\ell_{s}^{2}}=\frac{\alpha_{s}t}{\ell_{s}^{2}} \end{array} \end{array}$$

where Bi and Fo are the Biot and Fourier numbers, $F_{S}^{\mathrm{net}}$ is a net outwards entropy flow rate [J K${}^{-1}$ m${}^{-3}$] (11) and $F_{Q}^{\mathrm{net}}$ is a net outwards heat flow rate [J m${}^{-3}$], with subscripts cond denoting conduction, stor storage and *s* a solid. Bi represents competition between heat convection at the surface and conduction into the solid, while Fo represents competition between heat conduction into and storage of internal energy within the solid [147,150]. Similarly, applying (39) and (54) to a phase change yields:

(A9) $$\begin{array}{c} \begin{array}{cccc} {\hat{\Pi }}_{d/\alpha ,i}\; & =\frac{{\hat{\dot{\sigma }}}_{d}}{{\hat{\dot{\sigma }}}_{\alpha ,i}}=\frac{{\hat{\dot{\xi }}}_{d}|\Delta G_{d}|/T}{\alpha_{i}\rho_{i}c_{p,i}T^{2}\;||\nabla T^{-1}{\left|\right|}^{2}}\; & \; & \to {Ste}_{i}=\frac{|\Delta {\tilde{H}}_{\mathrm{lat},d}|/M_{d,i}}{c_{p,i}\Delta T} \end{array} \end{array}$$

where *d* is the reaction index, *i* is the index of the final phase, $\Delta {\tilde{H}}_{\mathrm{lat},d}$ is the molar enthalpy (latent heat) of the phase change [J mol${}^{-1}$], $M_{d,i}$ is the molar mass [kg mol${}^{-1}$] and ${Ste}_{i}$ is the phase Stefan or Jakob number (or its reciprocal), which assesses the competition between latent and sensible heat during a fluid–solid or gas–liquid phase change [81,181]. Note that $Ste_{i}$ is different for each direction of the phase change. For high-speed flow past a solid surface:

(A10) $$\begin{array}{c} \begin{array}{cccc} \Pi_{\mathrm{int},\mathrm{ad}}\; & =\frac{\left|\right|j_{S,\mathrm{ad}}\left|\right|}{\left|\right|j_{S}\left|\right|}=\frac{\left|\right|j_{Q,\mathrm{ad}}\left|\right|\frac{1}{T}}{\left|\right|j_{Q}\left|\right|\frac{1}{T}}∼\frac{{\left(\Delta T\right)}_{\mathrm{ad}}}{\Delta T}\; & \; & \to Ec=\frac{{\left(\Delta T\right)}_{\mathrm{ad}}}{\Delta T}=\frac{U_{\infty }^{2}}{c_{p}\Delta T},\\ \Pi_{\mathrm{int},\nu /\alpha }\; & =\frac{{\dot{\sigma }}_{\mathrm{int},\nu }}{{\dot{\sigma }}_{\mathrm{int},\alpha }}∼\frac{\rho \nu \ell U_{\infty }^{2}/T}{k\ell^{3}{\left||\nabla T|\right|}^{2}/T^{2}}∼\frac{\rho \nu U_{\infty }^{2}T}{k{\left(\Delta T\right)}^{2}}\; & \; & \to Br=\frac{\rho \nu U_{\infty }^{2}}{k\Delta T}=\frac{\nu U_{\infty }^{2}}{\alpha c_{p}\Delta T}=PrEc \end{array} \end{array}$$

where Ec and Br are the Eckert and Brinkman numbers, $U_{\infty }$ is the free-steam velocity [m s${}^{-1}$] and subscript ad indicates adiabatic. Ec compares the entropy flux or temperature difference to that of an adiabatic process, controlling the direction of heat transfer [93,147], while Br represents the ratio of macroscopic entropy production by viscous dissipation (71) to heat conduction (39) [58]. For wall heat transfer, the ratio of heat convection to inertial dispersion from (86) and (94) is:

(A11) $$\begin{array}{c} \begin{array}{cc} \Pi_{h_{Q}/I}\; & =\frac{{\dot{\sigma }}_{\mathrm{int},h_{Q}}}{{\dot{\sigma }}_{\mathrm{int},I}}=\frac{{\dot{\sigma }}_{\mathrm{int},h_{Q}}/{\dot{\sigma }}_{\mathrm{int},\alpha }}{{\dot{\sigma }}_{\mathrm{int},I}/{\dot{\sigma }}_{\mathrm{int},\alpha }}∼\frac{h_{Q}\Delta T/k||\nabla T||}{fPe_{\alpha }}\;\to \frac{1}{f}\;St\\ \mathrm{with}\;\;\; & St=\frac{Nu}{Pe_{\alpha }}=\frac{Nu}{RePr}=\frac{h_{Q}}{\rho c_{p}U}=\frac{h_{Q}\alpha }{kU} \end{array} \end{array}$$

where St is the Stanton number, often used instead of Nu. Applying the “Reynolds analogy” between momentum and heat transfer, this is correlated with *f*, Re and Pr [16,77,81,92,93,94,147,148].

Mass-transfer analogs of Bi, Fo and St, and a momentum-transfer analog of Fo, have also been defined [92,95,147,159].
